# Actin Filaments Couple the Protrusive Tips to the Nucleus through the I‐BAR Domain Protein IRSp53 during the Migration of Cells on 1D Fibers

**DOI:** 10.1002/advs.202207368

**Published:** 2023-01-25

**Authors:** Apratim Mukherjee, Jonathan Emanuel Ron, Hooi Ting Hu, Tamako Nishimura, Kyoko Hanawa‐Suetsugu, Bahareh Behkam, Yuko Mimori‐Kiyosue, Nir Shachna Gov, Shiro Suetsugu, Amrinder Singh Nain

**Affiliations:** ^1^ Department of Mechanical Engineering Virginia Tech Blacksburg VA 24061 USA; ^2^ Department of Chemical and Biological Physics Weizmann Institute of Science Rehovot 7610001 Israel; ^3^ Division of Biological Science Graduate School of Science and Technology Nara Institute of Science and Technology Ikoma 630‐0192 Japan; ^4^ Data Science Center Nara Institute of Science and Technology Ikoma 630‐0192 Japan; ^5^ Center for Digital Green‐innovation Nara Institute of Science and Technology Ikoma 630‐0192 Japan; ^6^ Graduate School of Brain Science Doshisha University Kyotanabe Kyoto 610‐0394 Japan; ^7^ Laboratory for Molecular and Cellular Dynamics RIKEN Center for Biosystems Dynamics Research Minatojima‐minaminachi Chuo‐ku Kobe Hyogo 650‐0047 Japan

**Keywords:** actin, cell forces, extracellular matrice nanofibers, IRSp53, membrane curvature, protrusions, stick‐slip migration

## Abstract

The cell migration cycle, well‐established in 2D, proceeds with forming new protrusive structures at the cell membrane and subsequent redistribution of contractile machinery. Three‐dimensional (3D) environments are complex and composed of 1D fibers, and 1D fibers are shown to recapitulate essential features of 3D migration. However, the establishment of protrusive activity at the cell membrane and contractility in 1D fibrous environments remains partially understood. Here the role of membrane curvature regulator IRSp53 is examined as a coupler between actin filaments and plasma membrane during cell migration on single, suspended 1D fibers. IRSp53 depletion reduced cell‐length spanning actin stress fibers that originate from the cell periphery, protrusive activity, and contractility, leading to uncoupling of the nucleus from cellular movements. A theoretical model capable of predicting the observed transition of IRSp53‐depleted cells from rapid stick‐slip migration to smooth and slower migration due to reduced actin polymerization at the cell edges is developed, which is verified by direct measurements of retrograde actin flow using speckle microscopy. Overall, it is found that IRSp53 mediates actin recruitment at the cellular tips leading to the establishment of cell‐length spanning fibers, thus demonstrating a unique role of IRSp53 in controlling cell migration in 3D.

## Graphical abstract

Using 1D suspended fibers capable ofrecapitulating 3D migration, the authros demonstrate that IRSp53 links protrusions to the nucleus through force generation to control 3D cell migration. IRSp53 losscauses reduced actin dynamics at cell tips and a loss of stress‐fiber‐based contractility thattransitions the stick‐slip migration to a sliding‐slower migratory mode due to uncoupling the nucleus from thecell body.

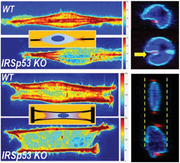

## 1 Introduction

Cell migration has been extensively studied using 2D surfaces with different topographies, as well as 3D matrix systems.^[^ ^1^ ^]^ Flat 2D migration assays have limited physiological relevance, while 3D matrices are intrinsically heterogenous, with local changes in matrix mechanics that can significantly alter migration patterns of a cell depending on the varieties in the local concentration and the degree of their cross‐linking. Additionally, in 3D gel matrices, it is exceedingly difficult to parse out the contribution of each constituent fiber during cell migration.^[^ ^2^ ^]^ Interestingly, key features of 3D cell migration were shown to be recapitulated through the use of narrow, 1D microprinted lines on a 2D surface,^[^ ^3^ ^]^ which are essentially patterns on a flat plane and are not real fibers. We have previously shown that suspended, 1D synthetic fibers of sub‐micron diameters coated with extracellular matrices (ECM) proteins result in protrusive, contractile, and migratory behavior that is sensitive to fiber curvature.^[^ ^4^ ^]^

Cell attachment and migration on the suspended 1D fibers begin with characteristic protrusions that coil around the fiber axis.^[^ ^4^, ^5^ ^]^ In 2D and 3D, filopodial protrusions can be mediated by the actin cytoskeleton and the inverse Bin/Amphiphysin/Rvs (I‐BAR) domain‐containing proteins.^[^ ^6^ ^]^ The I‐BAR domain proteins are crucial regulators of cell membrane curvature for protrusion formation, membrane trafficking, and cell migration, including cancer metastasis.^[^ ^7^ ^]^ Structurally, the intrinsically convex‐shaped surface of the I‐BAR proteins has been demonstrated to drive the extension of cell protrusions such as thin, “finger‐like” filopodia and broader, “sheet‐like” lamellipodia.^[^ ^8^ ^]^ In support of these experimental results, theoretical studies have also suggested that convex‐shaped proteins are key to forming cellular protrusions through the polymerization of cortical actin.^[^ ^9^ ^]^

Amongst the I‐BAR proteins, IRSp53 has been the most extensively studied member. It links the plasma membrane deformations and associated protrusive activity at the cell membrane to the underlying actin cytoskeleton,^[^ ^8^ ^]^ by binding to small GTPases, including Cdc42 and Rac1 during cell migration.^[^ ^10^ ^]^ IRSp53 also interacts with negatively charged lipids, including phosphoinositides such as phosphatidylinositol (3,4,5)‐trisphosphate, and phosphatidylinositol (4,5)‐bisphosphate,^[^ ^10^, ^11^ ^]^ where phosphoinositides are also able to activate Cdc42 and Rac1 through guanine nucleotide exchange factors. Especially, the kinases including PI‐3 kinase catalyzes the formation of phosphoinositides when cells are stimulated for migration.^[^ ^6c^ ^]^ The studies on flat 2D surfaces and 3D extracellular matrix gels have demonstrated that the depletion of IRSp53 results in impaired protrusive activity at the cell membrane.^[^ ^10^, ^12^ ^]^

In this study, we investigated whether the governing molecular machinery of cellular protrusions and cell migration discovered in the 2D systems and 3D gels can be extended to suspended 1D fibers. Using suspended fibers of 135 and 500 nm diameter, we probed the role of IRSp53 during cell spreading and migration on 1D fibers. We found that IRSp53 depletion resulted in reduced actin activity at the cell‐leading edges, leading to a reduction in overall cell contractility and changes in the migratory phenotype. These experimental results were confirmed by our theoretical model. Overall, our experimental and theoretical study in a biologically relevant 1D fibrous environment describes IRSp53's significant role in mechano‐transduction on 1D fibers, that is, the actin filament dynamics at the protrusive tips of elongated cells appears to be essential for cell migration and organization of stress‐fibers, which in turn control the overall cellular contractility and force transduction to the nucleus.

## 2 Results

### 2.1 IRSp53 Depletion Alters Spreading Dynamics on Suspended Fibers but not on Flat 2D

**Figure 1:**
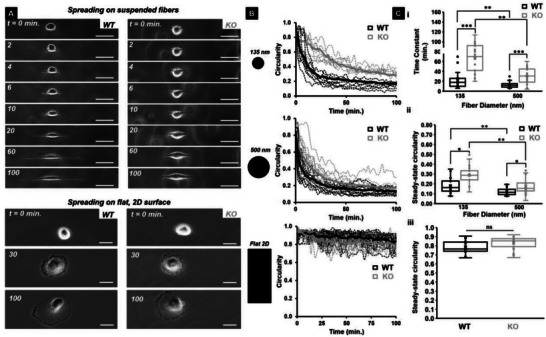
IRSp53 KO cells' circularity change is slower compared to WT cells on suspended fibers. A) Sequence of phase images showing WT cells (left panel) and KO cells (right panel) spreading on 135 nm diameter suspended fibers (top) and on the flat, 2D surface (bottom). Scale bars are 20 µm. B) Circularity profiles for both WT and KO cells on 135 and 500 nm diameter suspended fibers and on a flat, 2D surface. Black and grey dotted lines represent the individual circularity profiles for WT and KO cells respectively while the solid lines represent average profiles. C) Quantification of the i) time constant and ii) steady state circularity on both 135 and 500 nm diameter suspended fibers and iii) steady state circularity on the flat, 2D surface for both WT and KO cells. n = 20 for all categories on all substrates. All error bars shown represent the standard error of the mean.

In order to quantitate the role of IRSp53 at the interface of membrane dynamics and cytoskeletal contractility, we generated the IRSp53 knockout (KO) U251 glioblastoma cells (Figure S1, Supporting Information) using a CRISPR/Cas9 system, as described previously.^[^ ^13^ ^]^ We first inquired if IRSp53 depletion caused any effects easily observable under a microscope. We selected a cell‐spreading assay composed of suspended fibers of two diameters (high curvature ≈135 nm and low curvature ≈500 nm) spaced at least 20 µm apart to achieve spindle‐like elongated cell shapes attached to single fibers (**Figure** **1**A). We confirmed the fiber diameters using scanning electron microscopy (SEM) (Figure S2, Supporting Information). Cell spreading experiments on the suspended fibers were timed to capture spreading behavior from a rounded initial state (high circularity) to a more elongated state (low circularity) along fibers (Figure 1A,B). We calculated the area of cells as they spread and found that on the two diameters, both cell types spread at similar rates, and when compared with flat 2D, cells on suspended fibers had smaller areas (Figure S3, Movies M1,M2, Supporting Information). However, we observed that KO cells took longer times to achieve low circularity values on fibers (longer time constant) than the WT cells on both fiber diameters, while on flat 2D, both cell types remained highly circular during spreading (Figure 1C). Interestingly, between the two fiber diameters, we found that on smaller 135 nm diameter fibers, cellular movements and spreading were faster than that on larger 500 nm diameter fibers, and IRSp53 depletion resulted in a significant decrease of these. Overall, while the cellular area calculations were not able to distinguish major differences between the two cell types, the time‐scale of the circularity metric, driven by protrusive activity on the fibers, was able to clearly and quickly distinguish IRSp53 depletion effects.

### 2.2 IRSp53 Depletion Impairs Protrusion Dynamics and Coiling at the Tips of Protrusions

**Figure 2:**
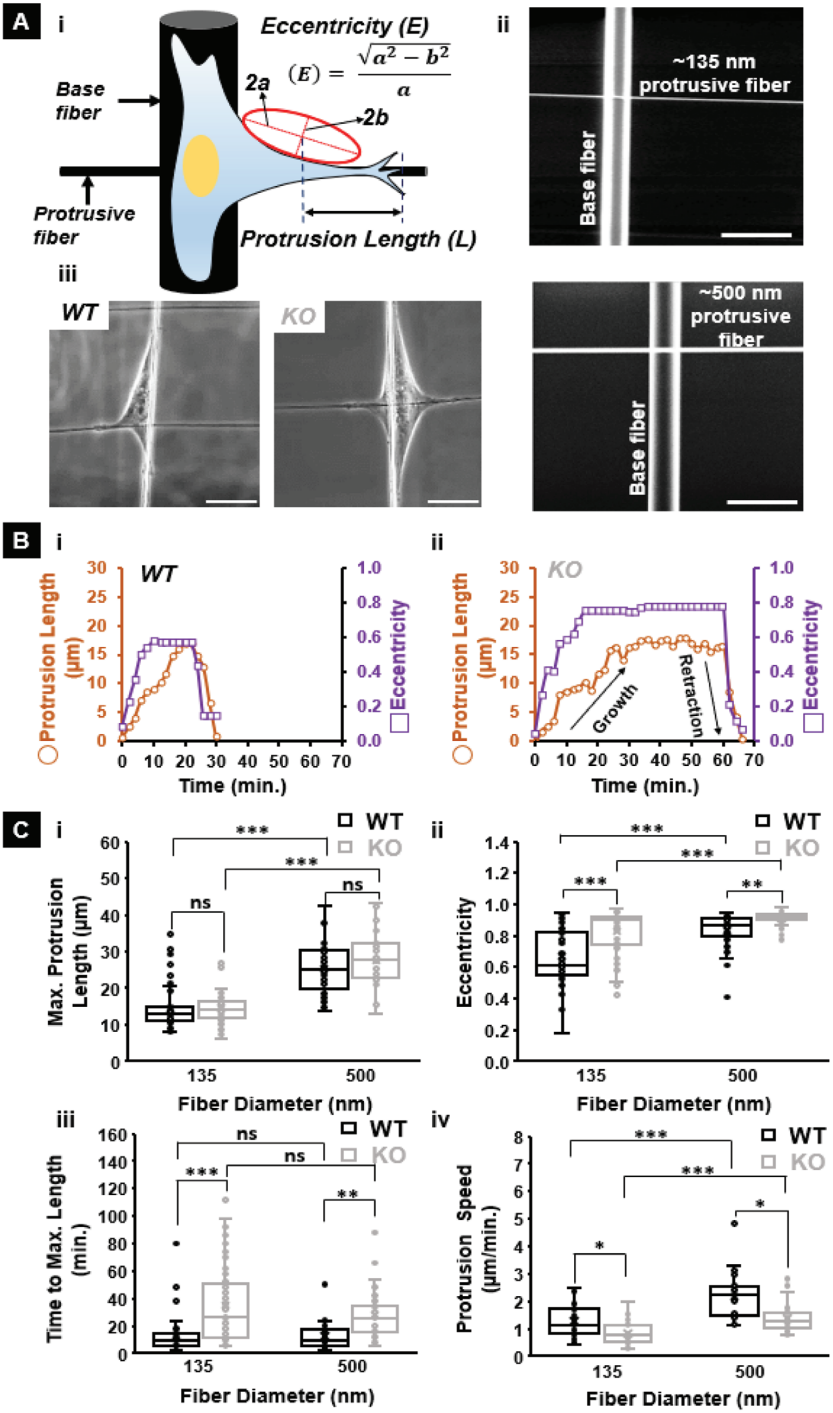
IRSp53 KO glioma cells extend protrusions slower compared to WT cells on suspended fiber networks. A) i) Schematic showing how the protrusion length and eccentricity are quantified on the fiber networks. ii) Scanning electron microscopy (SEM) images of the fiber networks manufactured using the non‐electrospinning STEP technique for the protrusion measurements. Scale bars are 5 µm. iii) Brightfield images depicting typical protrusions formed by a WT and KO cell on 135 nm diameter protrusive fibers. Scale bars are 20 µm. Representative protrusive cycles for both WT (B.i) and KO (B.ii) cells highlight the significant differences in protrusion formation dynamics. C) Quantifying the differences in i) maximum protrusion length, ii) eccentricity at the maximum protrusion length, iii) time taken to reach the maximum protrusion length and iv) protrusion speed between WT and KO cells on both 135 nm and 500 nm diameter protrusive fibers. n values for KO cells are 50 and 38 on 135 and 500 nm diameters respectively and for WT cells are 50 and 32 on 135 and 500 nm diameters respectively. All error bars shown represent the standard error of the mean.

Since cell spreading is initiated through the extension of protrusions, and IRSp53 KO cells were taking longer times to achieve lower circularities (elongated shapes) on suspended fibers of both diameters, we inquired if protrusion dynamics were affected by IRSp53 depletion. To quantitate protrusive activity, we used our approach of depositing large diameter (≈2 µm), suspended “base fibers” orthogonal to smaller diameter suspended “protrusive fibers” (**Figure** **2**A(i,ii)).^[^ ^4^, ^5^ ^]^ As previously reported, this configuration allows bulk cell body migration to be constrained along with the base fiber, while individual protrusive events are elicited and studied along the protrusive fibers. To quantify the protrusion dynamics, we measured the protrusion length (L) and the eccentricity (*E*, a measure of the protrusion width and shape at its base). Low eccentricity values (*E* < ≈0.6) signified “rod‐like” protrusions, while higher eccentricity values (*E* > ≈0.8) indicate broader, “kite‐shaped” protrusions. Using the combination of the protrusion length and eccentricity allowed us to quantitatively describe a “protrusion cycle.” A typical protrusion cycle commences with the rapid broadening of the protrusion at its base, that is, an increase in the eccentricity, followed closely by an increase in the protrusion length until the protrusion reaches a maximum length and finally retracts back to the main cell body (Figure 2B(i,ii), Movie M3, Supporting Information). We found no significant differences in the averages of maximum protrusion lengths between the KO and WT cells on both fiber diameters tested (Figure 2C(i)). However, we found that on both fiber diameters, the *E* was significantly higher for the KO cells suggesting broader protrusions (Figure 2C(ii)). Despite the maximum protrusion lengths being similar, we found that IRSp53‐KO cells took a longer time to reach them (Figure 2C(iii)), indicating lower protrusive speeds (Figure 2C(iv)). In line with our finding that KO cells took a longer time to achieve steady‐state circularity, the protrusive speeds on 135 nm diameter were significantly lower than on 500 nm diameter fibers. The eccentricity metric also showed a greater jump in the width of protrusions for KO cells on 135 nm compared to 500 nm diameters. Finally, we found that the KO cells exhibited significant fluctuations (extension and retraction) during their growth phase (Figure S4, Supporting Information).

**Figure 3:**
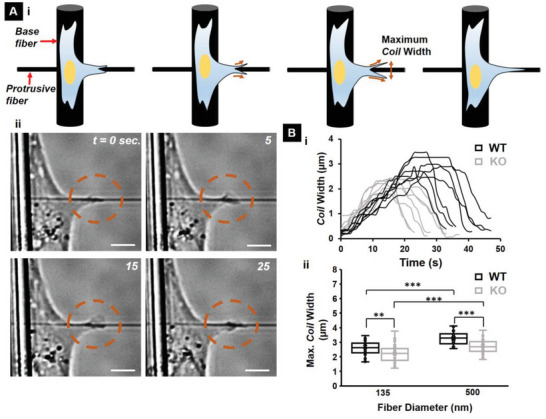
IRSp53 KO cells exhibited lower coil widths at the protrusion tip. A) i) Schematic showing a typical coiling cycle with ii) associated phase images for a KO cell on 135 nm diameter protrusive fiber. Scale bars are 5 µm each. Dashed orange circles highlight the coiling in each of the phase images. B) i) Representative coiling cycles for both WT and KO cells on 135 nm diameter protrusive fiber (7 representative profiles for each case). ii) maximum coil width highlighting the diminished coil width for the KO cells. n = 30 for both WT and KO cells on each of the two fiber diameters investigated. All error bars shown represent the standard error of the mean.

Previously, we have demonstrated that cells coil (wrap around the fiber axis) at the protrusion tip (**Figure** **3**A(i,ii)).^[^ ^4^, ^5^ ^]^ Based upon our findings of delayed protrusive activity in IRSp53 KO cells, we naturally inquired if these differences translated to differences in the coiling cycle occurring at the protrusion tips (Figure 3B(i), Movie M4, Supporting Information). While the maximum coil width increased with fiber diameter (Figure 3B(ii)), in agreement with our previous findings,^[^ ^4d^ ^]^ the maximum coil width was significantly lower for the KO case (Figure 3B(ii)). Overall, we found that IRSp53 protein depletion did not impact the protrusion lengths but impaired protrusion dynamics and led to diminished coil width at the protrusion tip.

### 2.3 IRSp53 Depletion Affects Actin Networks and Contractility

**Figure 4:**
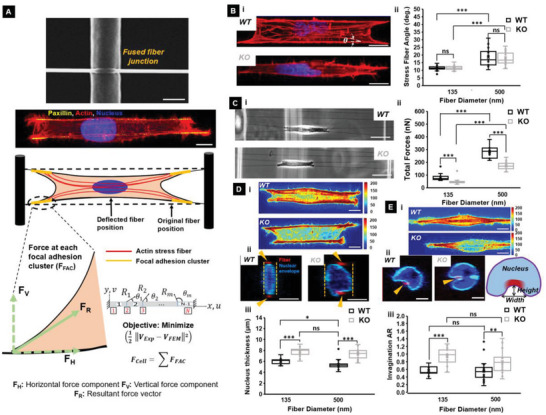
IRSp53 depletion leads to loss of contractility through loss of actin stress fibers A) Schematic providing an overview of how forces are calculated using fused nanonets. The SEM image shows a fused fiber junction. The scale bar is 2 µm. The fluorescent image shows actin filaments in red, nucleus in blue, and focal adhesion protein paxillin clustering in yellow. The scale bar is 20 µm. NFM establishes force vectors that originate from focal adhesion clusters and are directed along the actin stress fibers. An inverse finite element model minimizes the error between computational and experimental fiber deflections. B) Representative confocal fluorescence microscopy images of i) WT (top) and KO (bottom) cells with actin stained in red and ii) quantification of the stress fiber angles for both cell types on 135 and 500 nm diameter fibers. Scale bars are 10 µm. Dotted white lines in the fluorescent images depict the stress fiber angles. n values are 25 and 25 for the KO cells and 25 and 27 for WT cells on 135 and 500 nm diameter fibers respectively. C) Representative phase images of i) WT (top) and KO (bottom) cells exerting forces by pulling on suspended fibers with scale bars of 50 µm and ii) quantification of the forces exerted for both cell types on 135 and 500 nm diameter fibers. n values are 25 for both cell types on each of the two fiber diameters investigated. D) i) Representative heat maps in arbitrary units of the actin stress fiber distributions for the two cell types with scale bars of 10 µm. ii) Representative confocal images of WT and KO nucleus cross‐section (*yz* plane) with a scale bar of 5 µm, and iii) quantification of the nucleus thickness. E) i) Representative heat maps in arbitrary units of the actin stress fiber distributions for the two cell types attached to single fibers in spindle shapes with scale bars of 10 µm, ii) Representative confocal images of WT and KO nucleus cross‐section (*yz* plane) with a scale bar of 5 µm, and iii) quantification of the nucleus invagination AR. The schematic in (E.ii) shows how AR was measured. In the confocal images, the nucleus is in blue, the nuclear envelope is in cyan and the cross‐section of the suspended fibers is in red shown using yellow arrowheads. n values are 18 for the nucleus thickness measurements in (D) and 25 for the invagination AR measurements in (E) for both cell types on each of the two fiber diameters investigated. All error bars shown represent the standard error of the mean.

Since delayed cell spreading is associated with a loss of force exertion,^[^ ^14^ ^]^ we inquired if depletion of IRSp53 resulted in a loss of contractility. We used nanonet force microscopy (NFM) to quantify the forces exerted by single cells,^[^ ^4^, ^5^, ^15^ ^]^ as they spread on two parallel fibers of the same diameters (Movie M5, Supporting Information). Nanonet force microscopy (NFM) estimates forces by establishing force vectors that originate at focal adhesion clusters (FAC) and are directed along the actin stress fibers (**Figure** **4**A). On suspended fibers, cells form FAC at the poles (Figure S5, Supporting Information; 4 in case of cells spanning two parallel fibers); thus, the overall contractility of the cell estimated over the four FACs is *F_cell_* = ∑*FAC*. Using fluorescent images of filamentous actin, we first quantified the average stress fiber angle relative to the fiber (*θ*, Figure 4B(i)) and found no difference between the KO and WT cells on both fiber diameters (Figure 4B(ii)). For both cell types, the average stress fiber angle increased significantly with the increase in fiber diameter. However, we found that IRSp53 KO cells exerted ≈40% less force than WT cells (Figure 4C(i,ii)). This decrease in contractile force was presumably due to a reduction in the density of prominent, cell‐length spanning stress fibers in the IRSp53 depleted cells as compared to the WT cells on the two parallel suspended fibers (Figure 4D(i); Figure S6, Supporting Information), as well as on a single fiber (Figure 4E(i)). On quantifying the distribution of actin stress fiber lengths (normalized to the cell length) we found that in the KO case, regardless of fiber diameter, more than 70% of the actin fibers appear as small fragments (i.e., normalized length < 0.2) and <10% of the fibers span upward of 60% of the cell length. This is in stark contrast with the WT case, where almost 60% of stress fibers span upward of 60% of the cell length (Figure S7, Supporting Information). By comparison, the decrease in prominent stress fibers due to the depletion of IRSp53 was not observed on the flat 2D surface (Figure S8, Supporting Information). In contrast to the stress fiber density, we observed no significant differences in the distribution of focal adhesions in the WT and KO cells, on both the suspended fibers and on a flat 2D surface (Figure S9, Supporting Information).

Given that actomyosin contractility‐driven forces have previously been implicated in modulating nuclear deformations,^[^ ^16^ ^]^ we inquired if the diminished contractile forces exerted by the KO cells are manifested in modified nuclear compression. Using confocal microscopy, we measured the nucleus thickness for both KO and WT cells as reported by us earlier.^[^ ^15b^ ^]^ We found that the KO cells had ≈30% thicker nuclei (indicating reduced nucleus compression, Figure 4D(ii,iii)). To confirm that these results were not exclusive to U251, we quantified the stress fiber angles, the exerted forces, and the nucleus thickness for gingival cancer Ca9‐22 WT and KO cells (Figure S10, Supporting Information). Motivated by these results on two fiber systems, we enquired if there were any differences in the nucleus shape for cells migrating on single fibers. Confocal imaging revealed invaginations in the nuclear envelope at fiber‐specific sites.^[^ ^15b^ ^]^ We examined the shape of local invaginations and found a remarkable difference between the WT and KO cells (Figure 4E(i,ii)). The invaginations of the nucleus in the KO cells were sharper and deeper than the invaginations in the WT cells. Quantifying the invagination aspect ratio (height/width) revealed an increase in the invagination aspect ratio (AR) for the KO cell nuclei. Altogether, we found that the number density of cell‐spanning stress fibers was significantly diminished in the KO cells. Consequently, the KO cells exerted significantly lower forces on the external fibers than the WT cells, which ultimately translated to reduced nuclear compressions and invaginations at the nucleus‐fiber‐contact sites.

### 2.4 IRSp53 Depletion Causes Loss of Stick‐Slip Migration and a Breakdown in Nucleus‐Cytoskeleton Coupling

**Figure 5:**
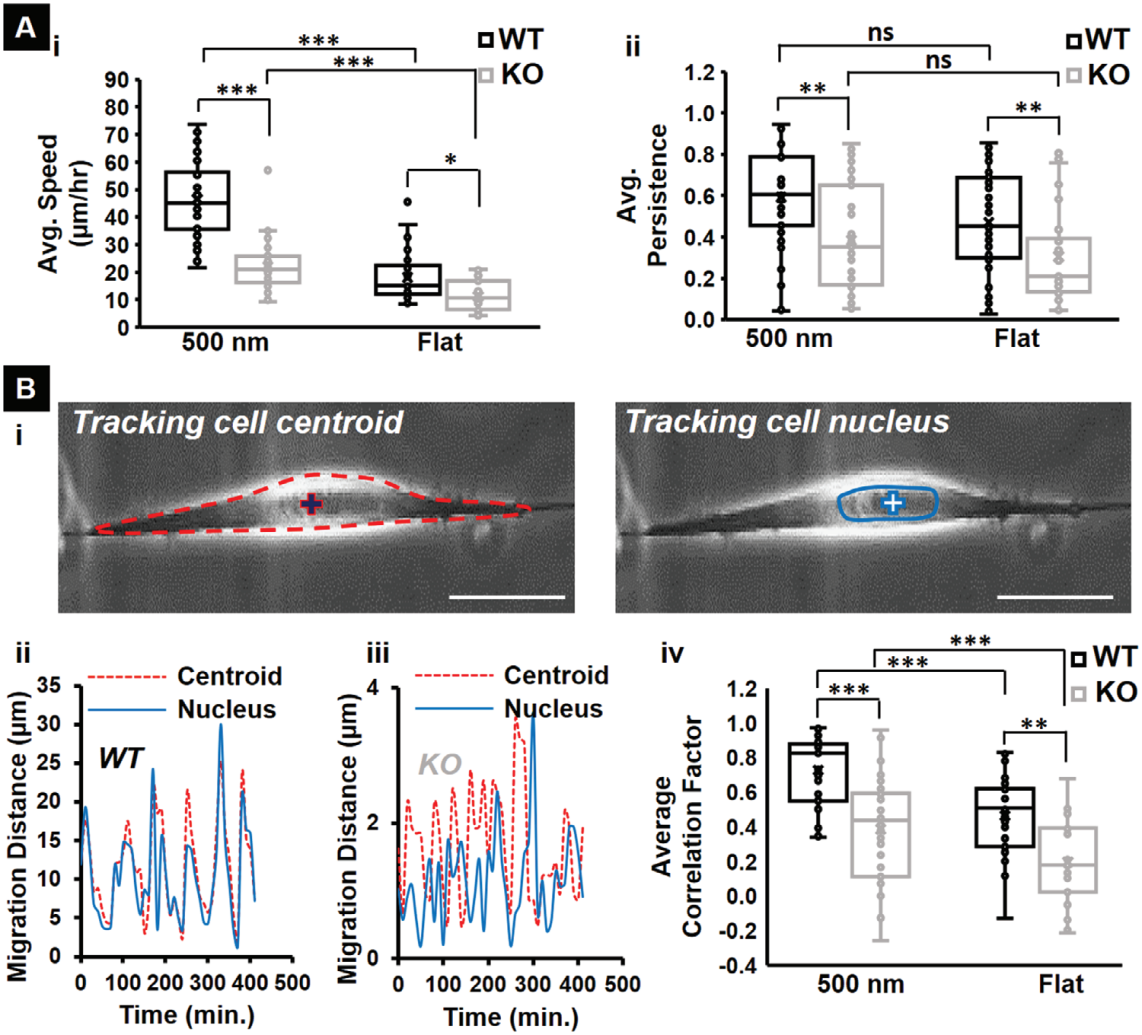
IRSp53 depletion leads to loss of nucleus‐cell body coupling and shift in migratory pattern. A) Quantification of the i) speed and ii) persistence of both WT and KO cells on 500 nm diameter suspended fibers and flat 2D surface (control). B) Quantifying the synchronicity between the nucleus and cytoskeleton during migration for WT and KO cells. i) Phase images showing that either the centroid or nucleus can be tracked during migration. The red “+” sign indicates the centroid of the cell body that is outlined by the red dashed boundary. The blue “+” sign indicates the nucleus of the same cell. Typical transient profiles of the distances migrated by the centroid (red) and nucleus (blue) for ii) WT and iii) KO cells. iv) Quantification of the average correlation factor between the centroid and nucleus movement during migration for both WT and KO cells on suspended fibers and flat surface. n values for both cell types are 35 on each substrate. All error bars shown represent the standard error of the mean.

Given that IRSp53 depletion altered protrusive behavior and force exertion, we inquired if these changes ultimately resulted in differences in migration dynamics. Thus, we quantified single‐cell migration of both KO and WT cells attached to 500 nm diameter fiber networks as well as on a flat 2D surface. We first characterized the morphology of migrating cells (Figure S11, Supporting Information), and as expected, both KO and WT cells showed significantly lower circularity and higher AR on fibers than the flat surface, in agreement with our previous findings.^[^ ^17^ ^]^ Next, we investigated the migration dynamics and found that WT cells migrated on both substrates using the stick‐slip mode (Movie M6, Supporting Information), whereby in a migration cycle, the leading edge would continue to grow, and the trailing edge would retract in a slingshot manner. In contrast, KO cells exhibited a slower, smooth, and sliding migratory behavior with lower persistence (**Figure** **5**A(i,ii)). In the stick‐slip mode of migration, WT cells demonstrated synchronous displacement between the bulk cell body and the nucleus, while in KO cells, the nucleus lagged the cell body displacement during migration. To further quantify this behavior, we measured the correlation factor between the nucleus and the cell body displacements, on short time scales of ≈5 min. The correlation factor ranges from −1 to 1 (see Experimental Section for details), with values close to 1 indicating highly correlated motion (i.e., the nucleus and cell body move in the same direction together), values close to zero indicating no correlation, and values close to −1 indicating anticorrelated motion. We found that both KO and WT cells on fibers had higher correlation values in general compared to their counterparts on flat 2D indicating a stronger coupling between the cell body and the nucleus during a migration cycle on the 1D fibers. However, depletion of IRSp53 resulted in a loss of correlation on both the fibers and flat substrate, with cells on a flat substrate having values closer to zero, indicating weak mechanical coupling between the nucleus and the cell body displacements on short timescales during migration (Figure 5B). In addition to the reduced coordination during migration, we also found that the KO cells exhibited fewer fluctuations in the cell shape during migration than the WT cells (Figure S12, Supporting Information). Overall, we found that cells moved faster and with higher persistence on fibers than on the flat substrates and that IRSp53 depletion resulted in the loss of stick‐slip migratory mode and reduced mechanical coupling between the nucleus and the cell body during migration.

### 2.5 Restoration of IRSp53 Recovered WT Behavior

**Figure 6:**
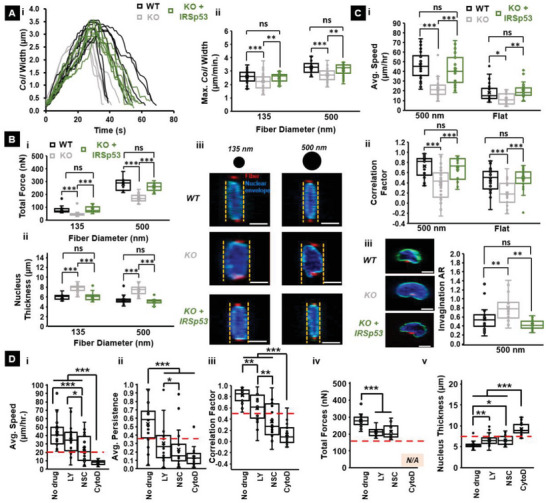
IRSp53 reconstitution in KO cells recovers WT cell function A) i) Representative coiling cycle profiles for IRSp53 KO, WT, and IRSp53 reconstituted cells on 500 nm diameter suspended fibers. Eight representative profiles were selected for each cell category. Quantification of ii) maximum coil width for all three cell types on both 135 and 500 nm diameter protrusive fibers. n values are 30 for KO cells, 30 for WT cells, and 35 for IRSp53 reconstituted cells on each of the two fiber diameters tested. B) Quantification of the i) total forces exerted and ii) nucleus thickness of IRSp53 KO, WT and IRSp53 reconstituted cells on both 135 and 500 nm diameter fibers. iii) Representative confocal images showing that reconstitution of IRSp53 in IRSp53 deficient cells leads to recovery of nucleus thickness similar to the WT cells. n values for the force calculations are 25 for all cell categories on both fiber diameters tested. n values for the nucleus thickness measurements are 18 for the KO cells on both fiber diameters, 18 for the WT cells on both fiber diameters, and 18 and 22 for the IRSp53 reconstituted cells on the 135 and 500 nm diameter fibers respectively. In the confocal images, the nucleus is in blue, the nuclear envelope is in cyan and the cross‐section of the suspended fibers is in red. Scale bars are 5 µm. C) Quantification of i) average migration rate and ii) correlation between nucleus and centroid for IRSp53 KO, WT, and IRSp53 reconstituted cells on 500 nm diameter suspended fibers. n values are 35 for all three cell categories. iii) Representative confocal images showing invagination of the nuclear membrane on 500 nm diameter fibers and associated quantification of the invagination AR. In the confocal images, the nucleus is in blue, the nuclear envelope is in cyan and the cross‐section of the suspended fibers is in red. Scale bars are 5 µm. n values are 25 for all three cell categories for the invagination AR quantification. D) Quantification of the i) average speed ii) average persistence iii) average correlation between nucleus and centroid iv) total force exerted and v) nucleus thickness for WT cells on 500 nm diameter suspended fibers under different pharmacological inhibitor conditions. Red dashed lines represent the corresponding magnitude for the KO cells for each of the metrics respectively. n values are 25 for each metric and each drug condition used. Note that in the case of Cytochalasin D, the total forces exerted are listed as N/A because the cells round up and don't exert any quantifiable deflection on the suspended fibers.

Next, we confirmed that the impaired coiling dynamics at the protrusion tip reduced contractility, and hindered migration dynamics were due to the depletion of IRSp53 by quantifying the effects of restoring IRSp53. We expressed IRSp53 in the IRSp53 depleted cells (KO cell line + IRSp53) and observed that these cells had a protrusive behavior similar to those of WT cells (**Figure** **6**A). Furthermore, KO + IRSp53 cells exerted similar forces, resulting in increased nuclear compression and recovery of nucleus thickness similar to WT cells (Figure 6B). Finally, reconstituted cells recovered stick‐slip migration dynamics and nuclear invagination ARs similar to that of the WT cells (Figure 6C). Altogether, we found that IRSp53 KO cells reconstituted with IRSp53 protein (KO + IRSp53 cell line) recovered WT functionalities.

### 2.6 Role of PI‐3 Kinase and Rac in 1D Migration and Nuclear Shape

IRSp53 sits at the nexus of a complex signaling cascade mediating interactions with the plasma membrane via Rho GTPases, including Cdc42 and Rac1 and actin nucleating factors.^[^ ^8^, ^18^ ^]^ Thus, we examined which components of this signaling pathway played a significant role in the dynamics of the cellular protrusions on 1D suspended fibers. To do this, we treated the WT and KO cells with the PI‐3 kinase (PI3K) inhibitor LY294002, which is involved in the regulation of the GTPases as well as the recruitment of IRSp53, and Rac1 inhibitor NSC23766, and the actin polymerization inhibitor cytochalasin D (Figure 6D).^[^ ^19^ ^]^ The cell migration speed of both WT and KO cells, as determined by the movement of the centroid of the cells (as in Figure 5), was decreased by treatment of cells with LY294002 and NSC23766 (Figure S13, Supporting Information for KO cells and Figure S14, Supporting Information for cells on flat). While KO cells had reduced migration persistence, the persistence of KO cells was further decreased by NSC23766 treatments. WT cells exhibited decreased persistence when treated with LY294002 and NSC23766. Treatment with Cytochalasin D of both cells abrogated the migratory response (Figure 6D(i,ii)). Furthermore, the correlation between the movement of the nucleus and that of the cell centroid in the WT cells was uncoupled by the LY294002 and NSC23766 treatments (Figure 6D(iii)). However, the correlation of the KO cells was not further reduced by treatment with LY294002 treatment but was reduced with NSC23766 treatment, presumably because IRSp53 is more dependent on the PI‐3 kinase signaling than Rac1 signaling. These results suggest that PI‐3 kinase and Rac1, as well as IRSp53, couple 1D migration and nuclear shape.

We also measured the total force and nucleus thickness in the presence of these drugs and found a considerable decrease in the force and a corresponding increase in the nuclear thickness with the drug treatments (Figure 6D). Furthermore, the treatment of the cells with the inhibitor of the actin filament turnover, cytochalasin D, resulted in the complete loss of speed, persistence, correlation, and force, as well as the increase of the nuclear thickness (Figure 6D(iv,v)), indicating the essential role of actin polymerization in the 1D signaling.

### 2.7 Theoretical Analysis of IRSp53 Effects in 1D Migration

**Figure 7:**
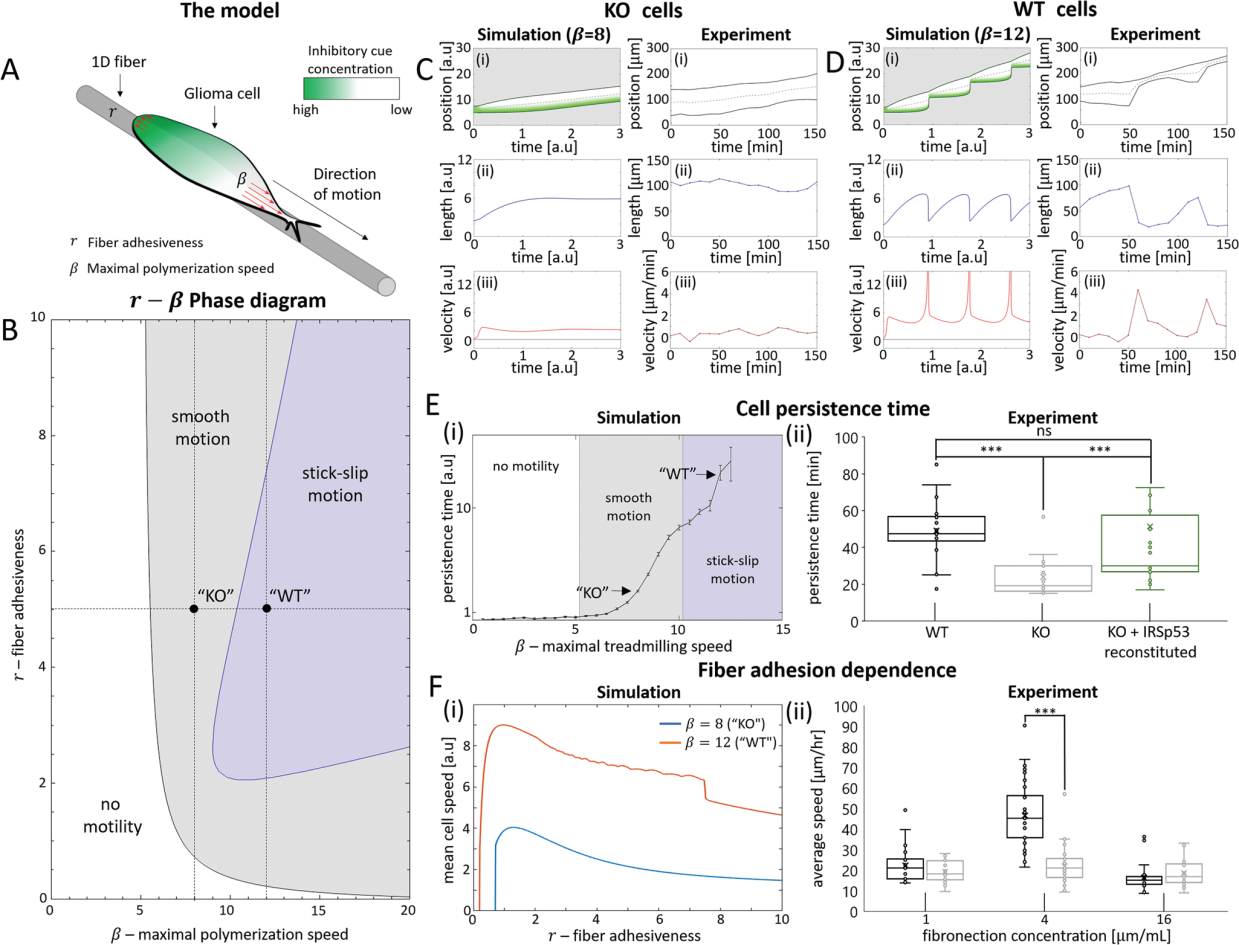
The theoretical model of 1D migration relates the migration patterns of the IRSp53 KO and WT cells on suspended fiber. A) Illustration of the theoretical model. A glioma cell migrating along a suspended linear 1D nanofiber. The motion of the cell is driven by actin polymerization (red arrows), which is inhibited in the presence of a polarity cue at the cell's edges (green/white colors indicate regions of high/low polarity cue concentration). *β* represents the maximal actin polymerization speed, and *r* represents the cell‐surface adhesiveness at the cell edges. B) The *r* − *β* phase‐diagram denoting the different migration patterns as function of the cell‐fiber adhesiveness (*r*) and the maximal actin polymerization speed (*β*). White/Gray/Blue colors indicate regions where the cell is non‐motile/migrating smoothly/exhibiting stick‐slip respectively. Dashed black lines indicate the cross sections of *β* = 12 and *r* = 5. C,D) Comparison between simulations and experiments of IRSp53‐KO cell and WT cells. C) Left panels display a simulation of a cell in the smooth regime (*β* = 8, *r* = 5) which reflect the behavior of the IRSp53‐KO cells (right panels). D) Left panels display a simulation of a cell in the stick‐slip regime (*β* = 12, *r* = 5) which reflect the behavior of the WT cells (right panels). The migration characteristics which are compared are: i) The kymographs of the cell migration. Black lines indicate the cell's edges. Green/White color in the simulation indicates a region of high/low inhibitory cue concentration. The gray dashed line indicates the position of the nucleus in the experiment and the mean geometric center in the simulation. ii) The cell's length, and iii) The cell's speed as a function of time. E) Cell persistence time in simulations and experiments: i) Simulations showing the persistence time as a function of the maximal polymerization speed (*β*) along the *r* = 5 cross‐section of the phase diagram (horizontal black dashed line in B). Arrows point to *β* = 8 and *β* = 12 which represent the IRSp53‐KO cells (C), and WT cells (D), respectively. White/Gray/Blue color indicates the region of no‐motility/smooth motion/stick‐slip motion in the model. ii) Experimental measurements showing the persistence time in WT cells (black), IRSp53‐KO cells (gray), and IRSp53 reconstituted KO cells (green). F) Fiber adhesion dependence in simulations and experiments: i) Simulation results showing how the mean velocity of the cell in our migration model varies as a function of the cell‐fiber adhesiveness (*r*) for two different values of maximal actin polymerization speeds (*β* = 8 representing the IRSp53‐KO cells and *β* = 12 representing the WT cells). ii) Quantification of the average migration rate for three different fibronectin concentrations (1, 4, and 16 µg ml^−1^) for both WT and IRSp53‐KO cells on 500 nm diameter suspended fibers. n = 35 for both cell types for the 4 µg ml^−1^ case and n = 28 for both cell types on the other two fibronectin concentrations.

Having found that IRSp53 depletion causes profound changes in morphology, force exertion, and migratory behavior, we inquired if we could describe the migratory behavior of IRSp53 KO cells using a simplified theoretical model of 1D cell migration.^[^ ^20^ ^]^ The model allows us to qualitatively relate robustly the observed changes in migration patterns to possible effects of IRSp53 on the migration mechanism. Within this model, the motion of a 1D glioma cell is driven by the actin polymerization activity at its two opposing ends (**Figure** **7**A). The actin polymerization activity is dictated by the advection‐diffusion transport of the polarity cue which inhibits actin polymerization when it is localized at the cell's edges.^[^ ^20^ ^]^

In the presence of the polarity cue, the maximal polymerization speed that the cell can assume at its leading edge is denoted by *β*. The cell‐fiber adhesiveness is also assumed to be localized at the cell's edges,^[^ ^21^ ^]^ and allows the edges to stick to the fiber, and thus allow the cell to elongate and vary its length when it is polarized. The average cell‐fiber adhesiveness is denoted by *r*.

We plot the *r* − *β* phase diagram of the different migration patterns for the glioma cell, as predicted by our model (Figure 7B). In the phase diagram, we find that a non‐motile phase occurs when either the cell‐fiber adhesiveness (*r*) is low, or when the maximal polymerization speed (*β*) is low (white region in Figure 7B). Beyond the non‐motile regime, for larger *r* or *β*, we predict a regime where cells migrate smoothly with an overall constant length (gray region in Figure 7B), and as *β* increases further, the cell is predicted to transition into stick‐slip migration mode (indigo region in Figure 7B). The model also predicts that the cell's speed increases as *β* increases.^[^ ^20^ ^]^

Comparing the theoretical model to the experimental observations of decreased protrusive speed and low force exertion, we can therefore propose that the IRSp53‐KO cells have a lower maximal actin polymerization speed (*β*) which places them in the slower and smooth migration regime (Figure 7C), while the WT cells have a higher level of *β*, placing them in the stick‐slip migration regime (Figure 7D).

The model, therefore, naturally explains the observed decrease in persistence in the IRSp53‐KO cells when compared to the WT cells (Figure 7E). IRSp53 is known to couple with the actin polymerization machinery, and we therefore propose to correlate it with the parameter *β*. With the increase of *β*, the speed of the actin polymerization increases, which in turn gives rise to a faster actin retrograde flow and a larger front‐back gradient of the polarity cue.^[^ ^22^ ^]^ Thus, cells with a higher speed of the internal actin flow have a higher persistence time, remaining polarized along one direction of motion (in 1D), despite various internal noise sources; a prominent result of the Universal Coupling of Speed and Persistence (UCSP) model.^[^ ^20^, ^22^ ^]^

We tested the validity of our model further using two orthogonal approaches. First, we experimentally altered the cell‐fiber adhesion parameter *r* by altering the concentration of fibronectin used to coat the synthetic suspended nanofibers and compared it to the predictions from the model (Figure 7F). We used three fibronectin concentrations: low concentration (1 µg ml^−1^), intermediate concentration (4 µg ml^−1^), and high concentration (16 µg ml^−1^). We observed a biphasic relationship between migration dynamics and the fibronectin concentration for both cell types, with the fastest speed obtained at the intermediate 4 µg ml^−1^ (Figure 7F(ii); Figure S15, Supporting Information).

### 2.8 Actin Flow Measurement by Lattice Light‐Sheet Microscope

**Figure 8:**
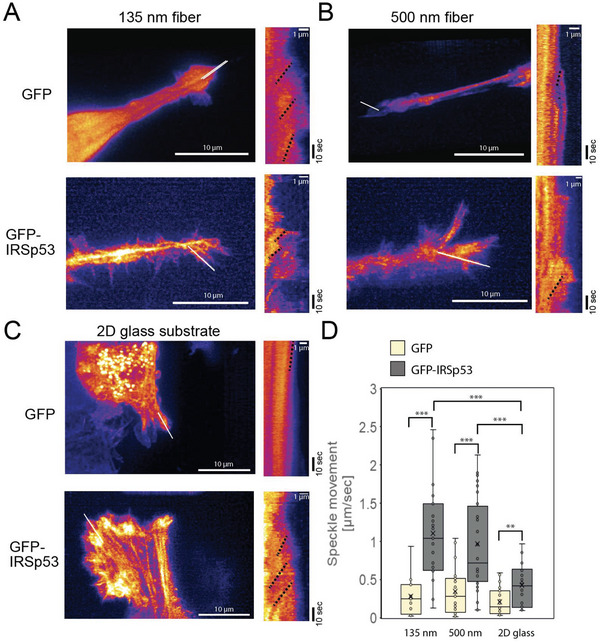
The Halo‐tagged actin flow in the IRSp53‐knockout cells expressing GFP or GFP‐IRSp53 on 1D fibers or 2D flat surfaces under a lattice light‐sheet microscope. The heat maps of Halo‐tagged actin fluorescence and their kymographs of the cells migrating on A) the 135 nm fiber, B) the 500 nm fiber, and C) the glass surface. The kymographs were generated by drawing a straight line at the cell's leading edge, as indicated by the white lines. The *x*‐axis of the kymograph represents the displacement (µm), whereas the *y*‐axis represents the time (seconds) over 102 s. The yellow dashed lines indicate the examples for the determination of actin flow rates. Scale bar 10 or 1 µm as indicated. D) Distribution of the actin flow rate with the mean ± SD. Each dot represents an individual measurement of the retrograde flow from one region of interest (ROI). One to three ROI per cell were measured. Statistical significance is shown by **p < 0.01; ***p < 0.001 by two‐sample equal variance *t*‐test.

We next tested the model prediction that WT cells have high actin polymerization at the leading edge by analyzing the retrograde flow of actin using the Halo‐tagged actin introduced into the WT and KO cells by using a lattice light‐sheet microscope that is suitable for imaging in 3D space. Using speckle tracking of the images obtained by lattice light‐sheet microscope,^[^ ^23^ ^]^ we generated actin flow kymographs correlating with the extent of the actin polymerization at the leading edge^[^ ^24^ ^]^ We found that the retrograde actin flow was faster in the IRSp53‐KO cells expressing IRSp53 than in the IRSp53‐KO cells expressing GFP, both for the 2D flat surface and the fibers (**Figure** **8**, Movies M7–M12, Supporting Information).

Overall, our theoretical 1D model of cell migration describes the transition of slip‐stick migratory behavior to a smooth low‐speed migratory mode in cells depleted of IRSp53, as well as their adhesion‐dependent speed variations, through a reduction of actin polymerization at the cell's leading edges, which we confirmed experimentally.

## 3 Discussion

In this study, we examined the molecular mechanism of 1D cell migration on suspended fibers. Because of the characteristic protrusions of the cells on the 1D fibers, we focused on IRSp53, which links the membrane to the actin cytoskeleton in cellular protrusions, in both 2D and 3D environments. We established a role of IRSp53 in mediating protrusive activity on the 1D fibers, where cells are forced to adapt to the elongated shape, and migrate in a “stick‐slip” pattern in U‐251 glioma cells and Ca9‐22 cells. IRSp53 was found to be essential for force generation during the 1D migration, presumably by affecting the actin filament formation at the edges of the cellular protrusions. IRSp53‐related actin polymerization activity at the cell edges is linked to the formation of cell‐spanning stress fibers that strongly affect the nuclear shape and its coupling to the cell movement during migration.

Previous studies on cell migration and IRSp53 have been primarily conducted on flat 2D substrates. During 2D and 3D cell migration, cellular protrusions occur by the reorganization of the actin cytoskeleton, which is under the control of small GTPases, including Cdc42 and Rac1. IRSp53 can couple the signals from both small GTPases to the membrane deformation and to the actin filament dynamics through WASP family proteins.^[^ ^10^, ^12^ ^]^ A recent study demonstrated that IRSp53 depletion leads to impaired migration in chemotaxis well‐plate assays through IRSp53 interaction with Cdc42 at its CRIB motif.^[^ ^10b^ ^]^ The SH3 domain of IRSp53 that interacts with actin nucleation promoting factors (NPFs), such as N‐WASP and the WAVE complex, and with VASP, which can also stimulate actin rearrangement, could offer an avenue via which IRSp53 could potentially affect actin polymerization recruitment to the membrane, thereby modifying both cytoskeletal contractility and migration dynamics.^[^ ^6^, ^10^, ^25^ ^]^ Therefore, the membrane recruitment of IRSp53, WASP family proteins, and VASP, as well as the activation of the small GTPases, are under the control of the phosphoinositide metabolisms, of which the activation of PI‐3 kinase is essential.^[^ ^6c^ ^]^

On the 1D fibers, our pharmacological studies indicated that the protrusive structure formation was dependent on Rac1 small GTPase, PI‐3 kinase, and actin polymerization, upstream and downstream factors of IRSp53. Interestingly, PI‐3 kinase dependency was absent, and Rac1 dependency was active in IRSp53 KO cells, but the overall migratory behavior was significantly decreased, suggesting that IRSp53 is a key mediator of this signaling machinery. Consistently, the IRSp53‐depleted cells also exhibit a decrease in actin polymerization at the cell leading edge on the 1D fibers (Figure 8). Therefore, we suggest that IRSp53 plays a similar role in regulating the cell migration machinery on 1D suspended fibers as previously described on flat 2D surfaces.

Interestingly, depletion of IRSp53 results in a stark decrease in cell‐spanning actin stress fibers (Figure 4B; Figure S7, Supporting Information), thus leading to a decrease in the overall contractile force exerted by the cell. However, it remains unclear how the IRSp53‐dependent actin filament assembly at the cell periphery resulted in the cell‐spanning actin stress fibers. One possibility is that NPFs are enriched in the periphery of the cells leading to the generation of de novo actin filaments which could be converted into the cell‐spanning actin bundles as had been suggested by the studies of stress fibers on 2D.^[^ ^26^ ^]^

Both peripheral actin polymerization and the contractility of the cells by the cell‐spanning actin network are key regulators of cell migration. In our study, IRSp53 depletion resulted in hindered migration dynamics in both cells migrating on 1D fibers and flat surfaces; however, the reduction in both speed and persistence of migration was more significant on the fibers, suggesting that cells moving on the fiber networks are more sensitive to the IRSp53‐dependent changes in actin dynamics not only at the leading edge of migration but also in the context of cell‐spanning actin filaments. This behavior may be due to the 1D fibers forcing the cells to be elongated, where the protrusive structures are naturally confined to the tips of the cells. Finally, we find that in such an elongated shape in the 1D environment, actin stress‐fibers that span from the tips of the cells to the nucleus play an important role in determining the nuclear shape and in mediating the coupling of the nuclear movements to those of the plasma membrane.

## 4 Conclusion 

In conclusion, our use of ECM‐mimicking suspended 1D fibers provides insights into possible 3D cell‐fiber interactions happening in vivo. We uncover the consequences of IRSp53 depletion for the cytoskeleton organization on 1D fibers, and the resulting effects on the traction forces exerted by the cells, cell shape, coupling of nuclei with cell body, and the mode of migration. Altogether, we provide new insights into the role of IRSp53 in mechanotransduction with implications in developmental, repair, and disease biology.

## 5 Experimental section

### Fiber Network Fabrication

The previously reported non‐electrospinning Spinneret Based Tunable Engineered Parameters (STEP) method was used to fabricate all the suspended fiber networks used in this study. Briefly, polystyrene (PS, Scientific Polymer Products, Ontario, NY) of ≈2 × 10^6^ g mol^−1^ molecular weight was dissolved in xylene (Thermo Fischer Scientific, Pittsburgh, PA) at 6% (w/w) concentration and 10% (w/w) concentration to prepare the solutions for spinning the ≈135 nm and ≈500 nm diameter fibers respectively. To spin the ≈2 µm base fibers for the protrusion, coiling, and force studies, a 5% (w/w) concentration solution of polystyrene of ≈15 × 10^6^ g mol^−1^ molecular weight was used. The solutions were prepared at least two weeks prior to spinning the fiber networks.

### Scanning Electron Microscopy

Environmental Scanning Electron Microscope was used to take images of the suspended fibers in order to confirm the fiber diameter. Prior to imaging the scaffolds, they were coated with a 7 nm thick layer of Platinum‐Palladium using a Leica sputter coater (Leica, Wetzlar, Germany). The images were taken at an electron beam voltage of 10 kV and a spot size of 3.5 using the Everhart‐Thornley detector. The working distance was maintained at ≈11 mm. An appropriate magnification factor was used depending on the application.

### Cell Culture and Drug Studies

U‐251 and Ca9‐22 cells were obtained from the Japanese Collection of Research Bioresources Cell Bank. The IRSp53 KO cells were generated by the CRISPR/Cas9 system, as described previously.^[^ ^13^ ^]^ The guide RNA targeting the first exon of IRSp53 (CCATGGCGATGAAGTTCCGG) was designed using the server http://crispr.mit.edu and inserted into the pX330 vector.^[^ ^13^ ^]^ After transfection, the cells were cloned by monitoring the GFP fluorescence from the reporter plasmid pCAG‐EGxxFP with the IRSp53 genome fragment using a fluorescence‐activated cell sorter [FACSAria (BD)].^[^ ^27^ ^]^ The expression of GFP or GFP‐IRSp53 in the IRSp53 knockout cells was performed by the retrovirus‐mediated gene transfer, as described previously.^[^ ^27^ ^]^ All cell lines were cultured in high glucose DMEM (Thermo Fisher Scientific) supplemented with 10% bovine calf serum (Thermo Fischer Scientific) and 1% penicillin‐streptomycin solution (Thermo Fischer Scientific) and stored in an incubator at 37 °C in 5% CO_2_ and humidified conditions. For the pharmacological inhibitor studies, cells were initially seeded as described previously on the nanofiber scaffolds. Subsequently, either 20 µm LY294002 (Millipore Sigma, St. Louis, Missouri for PI‐3 kinase), 75 µm NSC23766 (Millipore Sigma for Rac1), or 2 µm of Cytochalasin D (Millipore Sigma for Actin) was added to the cell culture media. The cells were incubated for 2 h. Following the incubation period, the cells were imaged as previously described.

### Cell Seeding and Experiment

In preparation for the experiments, the scaffolds were first affixed to the glass bottom of 6‐well dishes (MatTek Corp., Ashland, MA) using sterile, high‐vacuum grease (Dow Corning, Midland, MI). The scaffolds were then soaked in 70% ethanol for disinfection, followed by two phosphate‐buffered saline (PBS) washes (Thermo Fisher Scientific). Subsequently, the fibers were coated with either 4 µg ml^−1^ fibronectin (Invitrogen, Carlsbad, CA) or 4 µg ml^−1^ rhodamine fibronectin (Cytoskeleton Inc., Denver, CO) for 2 h prior to cell seeding to aid cell attachment to the fibers. For the quantification of how fibronectin concentration mediates cell migration, two additional fibronectin concentrations of 1 and 16 µg ml^−1^ were used. Once the cell culture reached ≈80% confluency, 0.25% Trypsin (ATCC, Manassas, VA) was added, and the culture was incubated for ≈5 min. After the cells detached from the flask surface, 3 ml of fresh cell media was added to dilute the effect of the trypsin. The entire solution was then placed in a centrifuge at 1000 RPM for 5 min. Following the centrifugation, the media was aspirated, and the cells were resuspended in fresh media. Finally, cells were seeded at a density of ≈3 000 000 cells ml^−1^ on the scaffolds.

### Immunostaining

Cells were fixed in 4% paraformaldehyde (Santa Cruz Biotechnology, Dallas, Texas), dissolved in PBS for 15 min, and rinsed in PBS twice. Three hundred microliters of permeabilization solution (0.1% Triton‐X‐100 in PBS) was then added to permeabilize the cells. After 15 min, the permeabilization solution was aspirated, and the cells were blocked using 10% goat serum in PBS for 30 min. Following this, the cells were incubated with either the anti‐lamin A/C primary antibody or the anti‐paxillin antibody (Abcam, Cambridge, United Kingdom) dissolved in antibody dilution buffer (0.3% Triton‐X‐100 and 1% BSA in PBS) at a ratio of either 1:200 (for lamin) or 1:500 (for paxillin) for either 2 h at 37 °C (for lamin) or 16 h at 4 °C (for paxillin). After the incubation period, the secondary antibody Alexa Fluor 647 goat anti‐mouse (Invitrogen, Carlsbad, CA) was diluted in the antibody dilution buffer at the ratio of 1:500 and added to the wells for lamin. For paxillin, the secondary antibody Alexa Fluor 488 goat anti‐rabbit (Invitrogen) was diluted in the antibody dilution buffer at a ratio of 1:350 and added to the wells. For imaging the filamentous actin for the stress fiber angle measurements, no primary antibody was added to the well. In this case, after permeabilization, rhodamine‐phalloidin (Abcam) was dissolved in the antibody dilution buffer at the ratio of 1:80 and added directly to the well. After immunochemistry, the samples were stored in a dark place for 45 min, followed by 3 PBS washes. Finally, the nuclei were counterstained with 300 nm of DAPI (Invitrogen) for 15 min. The scaffolds were kept hydrated in 2 ml of PBS and imaged using a 63× (water‐based immersion) magnification.

### Microscopy and Imaging

The cells were imaged using the AxioObserver Z.1 (with mRm camera) microscope (Carl Zeiss, Germany) at 20× for the protrusion, spreading, force, and migration studies and at 63× (water immersion objective) magnification for the coiling studies. The imaging intervals used were 5 min for the migration study, 3 minutes for the force and spreading studies, 2 min for the protrusion study, and 1 s for the coiling study. All the videos were analyzed using ImageJ (National Institutes of Health, Bethesda, MD).

For confocal microscopy of the immunostained samples, the cells were imaged using the LSM 880 confocal microscope (Carl Zeiss, Germany) at 63× (water immersion objective). The slice thickness was set to 0.36 µm for all the images, and appropriate laser powers were selected for the different lasers.

### Analysis of Biophysical Metrics

For the protrusion analysis, the maximum protrusion length was calculated as described previously. Briefly, the distance from the base fiber to the protrusion tip was first measured (*L_base_*). Subsequently, the largest possible ellipse was fit along the curvature of the protrusion such that one end of the ellipse coincided with a point on the protrusion that was at a distance of 0.8 × *L_base_* from the base fiber. Finally, the protrusion length (*L*) was measured as the distance from the tip of the protrusion to the projection of the intersection of the major and minor axes of this ellipse with the protrusive fiber. The eccentricity of the protrusion (*E*) was calculated as follows:

(1) $$Eccentricity=\frac{\sqrt{a^{2}-b^{2}}}{a}$$

where *a* and *b* are half of the length of the major and minor axis, respectively of the ellipse fit the protrusion (Figure S16, Supporting Information). The time taken to reach the maximum protrusion length was calculated as the total time taken from the first instance that a protrusion reached a threshold minimum length of 5 µm (this threshold was established in order to discount short‐lived membrane spikes and blebs) till the protrusion reached the maximum length. To calculate the average protrusion speed, the protrusion length was recorded for every frame (2 min). The instantaneous protrusion speed was first calculated as follows (in µm hr^−1^):

(2) $$Instantaneous\;Protrusion\;Speed=\frac{\left(L_{t}-L_{t-1}\right)\times 60}{2}$$

where *L*_*t* − 1_and *L_t_* is the protrusion length at any given frame and *L*_*t* − 1_ is the protrusion length in the previous frame. Finally, the average protrusion speed was calculated as the average of all the instantaneous protrusion speeds. To determine the percentage slope changes in the protrusion length during a protrusion cycle, the number of times the slope of the protrusion cycle changed sign from positive to negative was first recorded. This was then divided by the total number of time points in the protrusion cycle.

The coiling dynamics at the tip of the protrusion were calculated as previously described (Mukherjee et al., 2019). Briefly, the maximum coil width was calculated as the largest coil width during a coiling cycle. For the spreading analysis, the circularity of the cell was defined as follows:

(3) $$Circularity=\frac{4\times \pi \times Cell\;Area}{{\left(Cell\;Perimeter\right)}^{2}}$$

the value for circularity ranges from 0–1 wherein a value closer to 1 indicates a circular shape while a value closer to 0 indicates a more “straight‐line” shape. The steady‐state circularity was determined at ninety minutes after the initial seeding of the cells as there was no significant change in the circularity beyond this time point. The time constant for each circularity profile was calculated from the exponential decay equation shown below:

(4) $$Circularity\;\left(t\right)=Circularity\;\left(t=0\right)\times e^{\frac{-t}{\tau }}$$

where *τ* represents the time constant and is calculated as the time at which:

(5) $$Circularity\;\left(t\right)=\frac{1}{e}\times Circularity\;\left(t=0\right)$$

in order to generate the actin heat maps, fluorescent images of the actin stress fibers were first converted to grayscale using ImageJ. Subsequently, the greyscale images were imported into MATLAB and the function “colormap” was used to generate heat maps. The stress fiber length distribution analysis was performed using ImageJ and by considering only the linear sections of the stress fibers. A minimum length of 5 µm was used as a threshold based on previously reported lengths on flat 2D surfaces.^[^ ^28^ ^]^ For the FAC analysis, the FAC length was measured as the longest, continuous length of paxillin clustering. The FAC distance from the nucleus was measured to be the distance from the centroid of the nucleus to the starting point of the FAC length along the suspended fiber axis. The invagination ratio for cells suspended on single fibers was quantified from the yz nucleus mid‐plane projection obtained from confocal microscopy imaging as shown below:

(6) $$Invagination\;Aspect\;Ratio=\frac{Invagination\;height}{Invagination\;width}$$

for the migration analysis, cells were manually tracked using ImageJ, and the x,y location of the cell centroid was recorded for every second frame (i.e., every 10 min). The instantaneous speed was then calculated as follows (in µm hr^−1^):

(7) $$Instantaneous\;Speed=\frac{\sqrt{{\left(y_{t}-y_{t-1}\right)}^{2}+{\left(x_{t}-x_{t-1}\right)}^{2}}\times 60}{10}$$

where (*x*_*t* − 1_, *y*_*t* − 1_) are the coordinates of the centroid of a cell at any given frame in µm while (*x_t_*, *y_t_*) are the coordinates of the centroid of the same cell three frames (i.e., twelve minutes) later. The overall average speed of the cell was then calculated as the average of all the instantaneous speed values. The persistence of migration was calculated as follows:

(8) $$Persistence=\frac{\sqrt{{\left(y_{final}-y_{initial}\right)}^{2}+{\left(x_{final}-x_{initial}\right)}^{2}}}{\sum_{t=1}^{t=n}\sqrt{{\left(y_{t}-y_{t-1}\right)}^{2}+{\left(x_{t}-x_{t-1}\right)}^{2}}}$$

where (*x_final_*, *y_final_*) are the coordinates of the centroid of a cell at the last frame (nth frame) tracked in µm while (*x_initial_*, *y_initial_*) are the coordinates of the centroid of the same cell at the first frame tracked. The denominator was defined similar as above for the instantaneous speed. The correlation factor used for determining nucleus‐centroid coupling during migration was calculated as follows:

(9) $$Correlation\;Factor=\frac{Cov\left(centroid\;distance,\;nucleus\;distance\right)}{\sigma_{centroid\;distance}\sigma_{nucleus\;distance}}$$

where *Cov*(*centroid* *distance*, *nucleus* *distance*) represents the covariance of the centroid distance and nucleus distance and *σ* represent the standard deviation. The Shapiro‐Wilks normality test was used to ensure the normal distribution of data before calculating the correlation factor. To determine the average change in the area during the migration period, the magnitude of the difference in the area was calculated between every two frames (i.e., 10 min), and the average of all these differences was taken. A similar approach was followed to calculate the average changes in both perimeter and circularity.

### Force Model for Nanonet Force Microscopy

In order to quantify the forces from the fiber deflections, the fiber deflection was tracked for three randomly selected, consecutive frames and was analyzed in MATLAB (2017a) using the previously reported methods.^[^ ^4^, ^15^, ^29^ ^]^ Briefly, the ≈135 nm or ≈500 nm diameter horizontal force fibers were modeled as beams with fixed‐fixed boundary conditions as they were fused to a larger diameter, strut‐like, vertical base fibers at either end. Force vectors were established at FAC clusters at the poles and directed along the actin stress fibers. A custom finite element model was used to obtain the force fiber deflection profile based on an arbitrary initial force input. Subsequently, the error between the fiber profile predicted by the model and the experimentally tracked fiber profile was minimized using an optimization frame while simultaneously updating the force values iteratively. The average force was finally calculated as the average of the three consecutive frames selected.

### Theoretical Model of 1D Cell Migration

To model the cell migration of a single U251 glioblastoma cell on a linear fiber, the model developed by Ron et al., 2020 was used.^[^ ^20^ ^]^ In this model, the actin polymerization and adhesion dynamics were localized at the edges of the cell. For each edge, therefore, three dynamical equations were obtained: 1) For the position of the edge *x_i_*, 2) for the polymerization speed *v_i_*, and 3) for the adhesion concentration *n_i_*, where *i* denotes the front or the back

(10) $${\dot{x}}_{f,b}=\frac{1}{\Gamma_{f,b}}\;\left(\pm \left(\frac{r}{r+r_{0}}\right)v_{f,b}\mp k\left(x_{f}-x_{b}-1\right)\right)$$

(11) $${\dot{n}}_{f,b}=r\left(1-n_{f,b}\right)-n_{f,b}\exp\left(\frac{-\left(\frac{r}{r+r_{0}}\right)v_{f,b}+k\left(x_{f}-x_{b}-1\right)}{f_{s}n_{f,b}}\right)$$

(12) $${\dot{v}}_{f,b}=-\delta \left(v_{f,b}-v_{f,b}^{*}\right)$$

where *r* represent the cell‐fiber adhesiveness due to the binding and unbinding of slip‐bond adhesions at the cell rear, and the parameter *r*_0_ represents the fiber adhesiveness due to the binding and unbinding of catch‐bond‐like adhesions at the cell front. The parameter *k* represents the cell elasticity (mean cell spring constant). The parameters *f_s_* and *κ* are associated with the mechanical properties of the adhesions.^[^ ^20^ ^]^ The parameter *δ* represents a time scale for changes in the local actin polymerization speed.

The term Γ_*f*,*b*_ represents the friction term which changes with respect to the direction of motion of the cell's edge, and is given by

(13) $$\Gamma_{f,b}=\left\{\begin{array}{c} \;\frac{r}{r+r_{0}},\;\pm {\dot{x}}_{f,b}>0\\ n_{f,b}\kappa \exp\left(\frac{\left(\frac{r}{r+r_{0}}\right)v_{f,b}-k\left(x_{f}-x_{b}-1\right)}{f_{s}n_{f,b}}\right),\pm {\dot{x}}_{f,b}<0 \end{array}\right.$$

such that the top function in Equation (13) applies to edges that extend outward, while the complex lower function in Equation (13) applies to edges that retract.

The terms $v_{f,b}^{*}$ represent the steady‐state polymerization speeds at the cell's edges, and are given by

(14) $$v_{f,b}^{*}=\beta \left(\frac{1}{1+c_{f,b}}\right)$$

where *β* is the maximal actin polymerization speed which couples the steady‐state actin polymerization speed to the saturated polarity cue at the cell's edge.

The functional form of $c_{f,b}$ is given by

(15) $$c_{f,b}=\frac{c\left(v_{f}^{*}-v_{b}^{*}\right)}{D}\left(\frac{e^{-\frac{\left(v_{f}^{*}-v_{b}^{*}\right)x_{f,b}}{D}}}{e^{-\frac{\left(v_{f}^{*}-v_{b}^{*}\right)x_{b}}{D}}-e^{-\frac{\left(v_{f}^{*}-v_{b}^{*}\right)x_{f}}{D}}}\right)$$

where *D* is the diffusion coefficient of the polarity cue, and  *c* is a dimensionless quantity that encapsulates the concentration and its saturation properties.^[^ ^20^ ^]^ Equation (15) is derived from the description of the advection‐diffusion process of the polarity cue along the cell and is fully discussed in Maiuri et al. 2015^[^ ^22^ ^]^, and Ron et al. 2020^[^ ^20^ ^]^.

For the construction of the phase diagram (Figure 7B) two bifurcation curves were calculated:

1. The transition between the “no‐motility” and the “smooth motion” phases, calculated by finding a critical coupling strength *β*_*c*_ at which the actin polymerization speed is sufficient for the cell to become polarized. The value of *β*_*c*_ is determined by equating the two polarization lengths which are derived from the model.^[^ ^20^ ^]^

The first critical length arises due to the advection of the polarity cue

(16) $$l_{c}=\frac{c}{\sqrt{\frac{c\beta }{D}-1}}$$

and the second critical length is derived from the force balance between the actin polymerization and the cell elasticity

(17) $$l_{p}=\frac{1}{2}\left(1-c\right)+\frac{\beta }{2k}\left(\frac{r}{r+r_{0}}\right)+\sqrt{c+{\left(\frac{1}{2}\left(1-c\right)+\frac{\beta }{2k}\left(\frac{r}{r+r_{0}}\right)\right)}^{2}}$$

Equating the lengths in these two equations, the analytical form of *β*_*c*_ as a function of *r* is given by

(18) $$\beta_{c}\left(r\right)=\frac{D}{2c}+ck\left(1\;+\;\frac{r_{0}}{r}\right)+\frac{ck^{2}}{2D}{\left(1\;+\;\frac{r_{0}}{r}\right)}^{2}+\left(\frac{D\;-\;ck\left(1\;-\;\frac{r_{0}}{r}\right)}{2cD}\right)F\left(r\right)$$

(19) $$F\left(r\right)=\sqrt{{\left(D+ck\left(1+\frac{r_{0}}{r}\right)\right)}^{2}+4c^{2}Dk\left(1+\frac{r_{0}}{r}\right)}$$

2) The second transition line between the smooth motion and the stick‐slip motion is a Hopf bifurcation transition line which is obtained using a continuation method with AUTO07P.^[^ ^30^ ^]^

To calculate the persistence time in (Figure 7E(i)), random Gaussian noise was added to the actin polymerization speed (Equation (12)). The average amplitude of the noise was chosen to be Δ*v* = 2 (in dimensionless units, as *β*) to provide sufficient fluctuations for the cell to change its direction. The formula for the persistence time is given by

(20) $$\tau_{persistence}=\frac{\sum Times\;between\;direction\;changes}{Number\;of\;direction\;changes}$$

Throughout the simulations, the fixed parameters that were used in the model are *c* = 4, *D* = 4, *k* = 1, *f_s_* = 5, *κ* = 20, *r*_0_ = 1, *δ* = 100, *r* = 5. For Figure 7C,D the values that were used for the maximal actin polymerization speeds are *β* = 8, 12 (with respect to the black points in Figure 7B).

To calculate the cell's speed as a function of the adhesion parameter *r* in Figure 7F(i), the velocity of the moving front was averaged over 500 simulations with a very small noise amplitude of $\Delta v=10^{-7}$. We choose the velocity of the moving front as a proxy for the mean cell speed as it remains constant during stick‐slip events. This calculation was performed for *β* = 8, 12 (“KO”, “WT” in Figure 7B).

### Actin Flow Measurements by Lattice Light‐Sheet Microscope

The Halo‐tagged actin was introduced into the cells by a retrovirus and the cells were labeled by 5 nm Halo‐TMR (Promega) for 1 h for sparse labeling.^[^ ^31^ ^]^ After replacing the medium and culturing for 4 h, the cells were imaged with the Lattice light‐sheet microscope built in the Mimori‐Kiyosue laboratory at RIKEN Center for Biosystems Dynamics Research following the design of the Betzig laboratory^[^ ^23a^ ^]^ as described previously.^[^ ^23^, ^32^ ^]^ Cells were observed in a CO_2_ environment at 37 °C. After identification of the leading edge by IMARIS software, the images were exported to ImageJ software in the observation plane. To determine the velocity of the retrograde flow, a perpendicular straight line was drawn on the leading edge and the time‐lapse image sequences were analyzed by kymographs using ImageJ. Moving actin features were visualized in the kymographs as streak lines. The velocity of the flow was then obtained from the slopes of these lines.

### Statistics

Statistical analysis of the data was conducted using RStudio (RStudio, Boston, MA) software. The Shapiro‐Wilks normality test was used to check the normality of the data sets. Analysis of variance was used to test for the statistical significance between different data sets. The following symbols are used to represent the statistical significance levels: *<0.05, **<0.01, and ***<0.001. If there is no comparison shown between any data sets, it implies that there is no statistically significant difference between them. All the error bars represent the standard error of the mean. Data was acquired from multiple, independent sets of experiments.

## Conflict of Interest

The authors declare no conflict of interest.

## Author Contributions

A.S.N. conceived and supervised the research. A.S.N., S.S., B.B., and N.G. designed research. A.M. conducted experiments. H.T.H., T.N., Y.M.K., K.H.S., and S.S. generated the specific cell lines and performed actin flow measurements. J.R. and N.G. developed and implemented the theoretical model of 1D cell migration. A.M., A.S.N., B.B., S.S., J.R., and N.G. analyzed data. A.M. wrote the manuscript. All authors contributed to the editing of the manuscript.

## Supporting information

Supporting Information

Supplemental Movie 1

Supplemental Movie 2

Supplemental Movie 3

Supplemental Movie 4

Supplemental Movie 5

Supplemental Movie 6

Supplemental Movie 7

Supplemental Movie 8

Supplemental Movie 9

Supplemental Movie 10

Supplemental Movie 11

Supplemental Movie 12

## Data Availability

The data that support the findings of this study are available from the corresponding author upon reasonable request.
