# Supplementary material for: Actin Filaments Couple the Protrusive Tips to the Nucleus through the I‐BAR Domain Protein IRSp53 during the Migration of Cells on 1D Fibers
Source: Adv Sci (Weinh). 2023 Jan 25;10(7):2207368. doi: 10.1002/advs.202207368 (PMC9982589; doi:10.1002/advs.202207368)
Supplement: Supplementary file 1 — Supporting Information [file ADVS-10-2207368-s002.pdf]

ADVANCED SCIENCE
Open Access

Supporting Information

for *Adv. Sci.*, DOI 10.1002/advs.202207368

Actin Filaments Couple the Protrusive Tips to the Nucleus through the I-BAR Domain Protein IRSp53 during the Migration of Cells on 1D Fibers

Apratim Mukherjee, Jonathan Emanuel Ron, Hooi Ting Hu, Tamako Nishimura, Kyoko Hanawa-Suetsugu, Bahareh Behkam, Yuko Mimori-Kiyosue, Nir Shachna Gov, Shiro Suetsugu and Amrinder Singh Nain**

Supporting Information

Actin filaments couple the protrusive tips to the nucleus through the I-BAR domain protein IRSp53 during migration of cells on 1D fibers

Apratim Mukherjee, Jonathan E. Ron, Hooi Ting Hu, Tamako Nishimura, Kyoko Hanawa-Suetsugu, Bahareh Behkam, Yuko Mimori-Kiyosue, Nir S. Gov, Shiro Suetsugu, and Amrinder S. Nain**

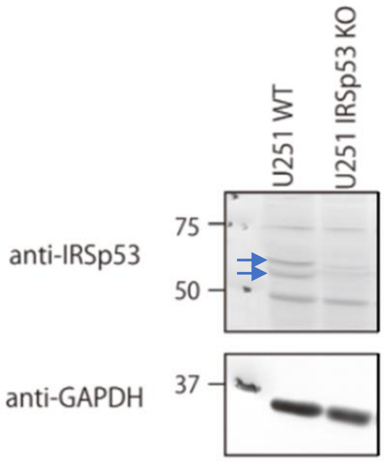

Supplementary Figure S1: Western-blot demonstrates IRSp53 knock-out in U-251 cells. Bands of IRSp53 are shown by blue arrows.

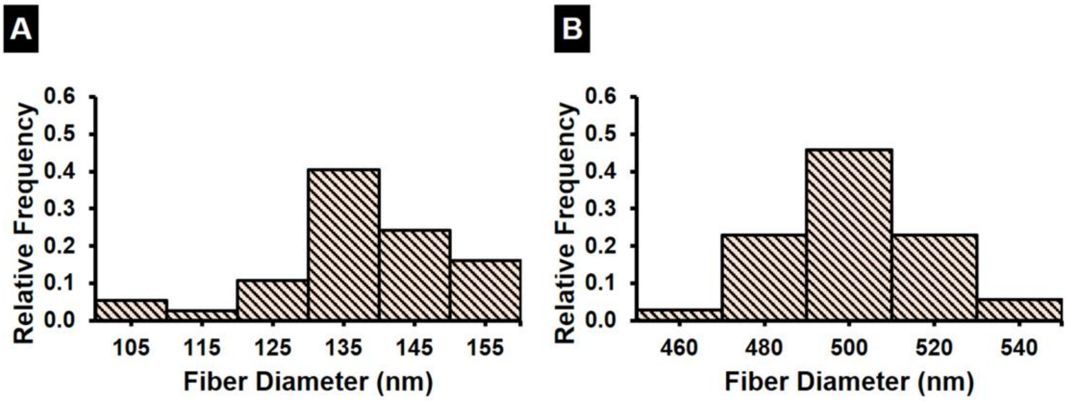

Supplementary Figure S2: Quantifying fiber diameter distribution. Histograms showing the fiber diameter distribution for (A) ~135 nm diameter, (B) ~500 nm diameter fibers used in this study. n values are 40, and 35, for the ~135 nm, and 500 nm diameters respectively.

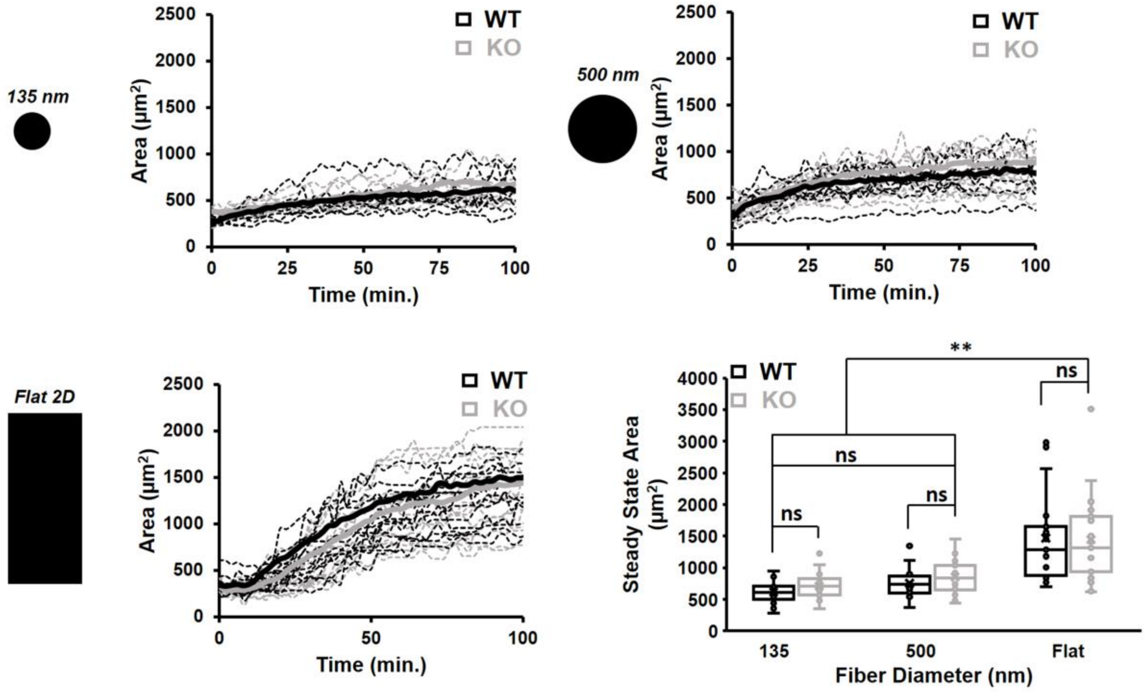

Supplementary Figure S3: Cell Spreading on suspended fibers. Area calculations as cells (WT (black) and IRSp53 KO (grey)) spread on suspended fibers and flat control 2D. Steady state cell area on fibers is less than 2D. n = 20 for each cell category. All error bars shown represent standard error of mean.

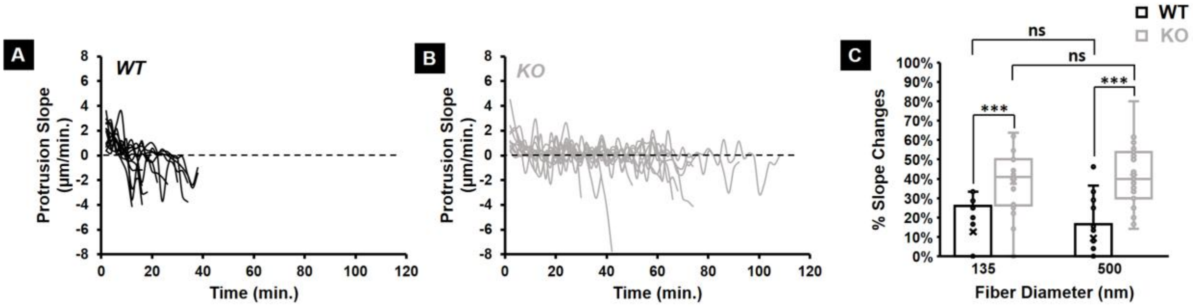

Supplementary Figure S4: Quantifying the fluctuations in protrusion cycle for both KO and WT cells. Representative protrusive cycle slopes for both (i) WT and (ii) KO cells (12 profiles are shown here for each cell type on 135 nm diameter protrusive fibers) showing significantly more fluctuations in the KO case. (C) Quantification of the percentage of slope changes for WT and KO cells on both 135 nm and 500 nm diameter protrusive fibers. n values are 27 for both WT and KO cells on each fiber diameter. All error bars shown represent standard error of mean.

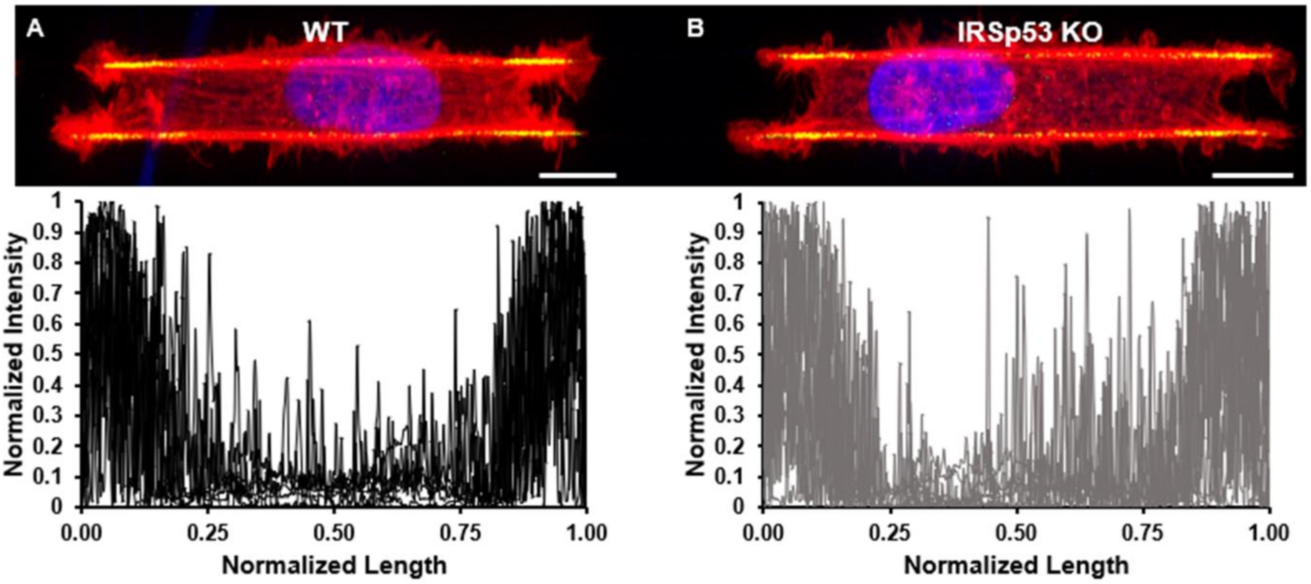

Supplementary Figure S5: Quantifying the Paxillin distribution for both WT and KO cells on suspended parallel fibers. Normalized intensity profiles for Paxillin distribution along the cell length for (A) WT and (B) KO cells elongated on suspended parallel fibers. High intensity at the ends of the cell lengths indicates the clustering of Paxillin into focal adhesion clusters (FACs) at the cell edges (yellow). Profiles are obtained from 15 cells for each category. Scale bars are 10 µm.

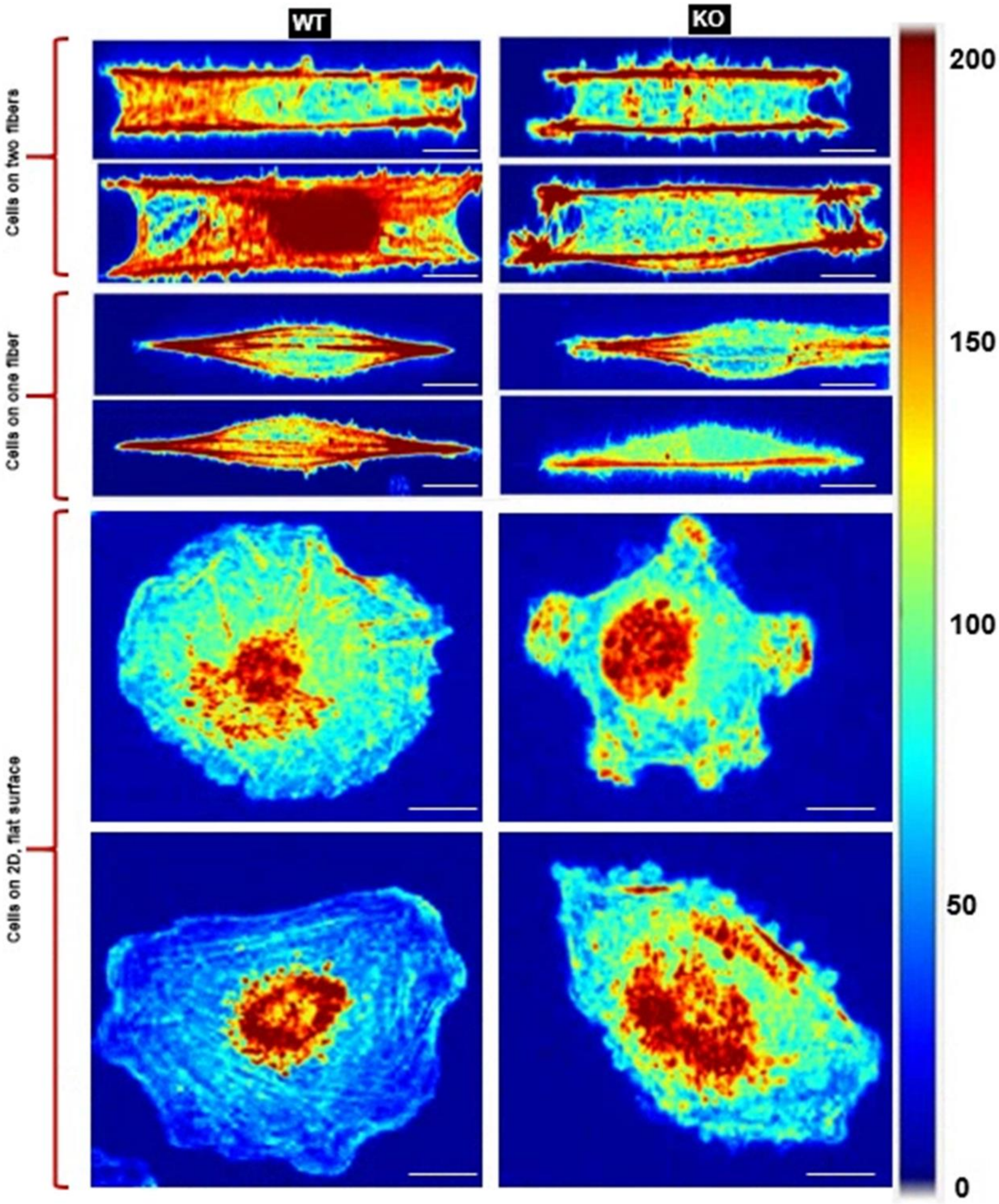

Supplementary Figure S6: Actin stress fiber distribution. Color maps (arbitrary units) showing the actin stress fiber distribution for both WT and KO cells on 500 nm diameter suspended fibers in cells attached to two fibers (top two panels), single fibers (middle two panels) and on flat, 2D (bottom two panels). Scale bars are all 10 µm. In all cell shapes, KO cells have reduced number density of actin stress fibers.

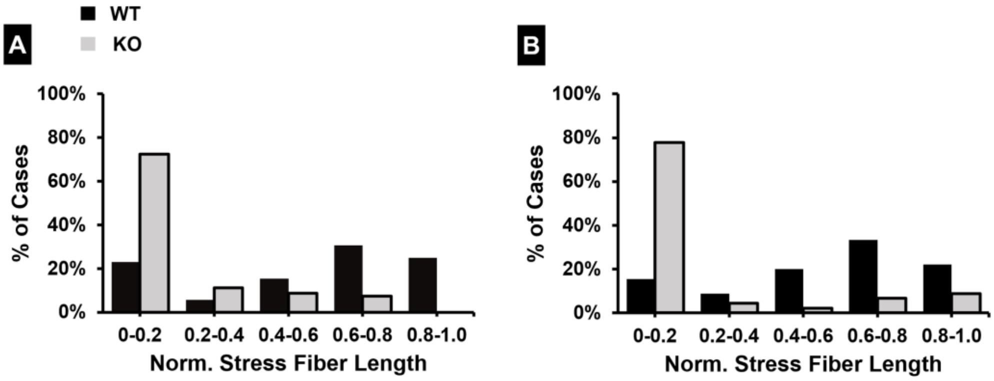

Supplementary Figure S7: Distribution of actin stress fiber lengths for (A) 135 nm diameter and (B) 500 nm diameter fiber cases. The stress fiber length was normalized to the cell length. n = 50 and 45 for both WT and KO cells on 135 nm and 500 nm diameter respectively.

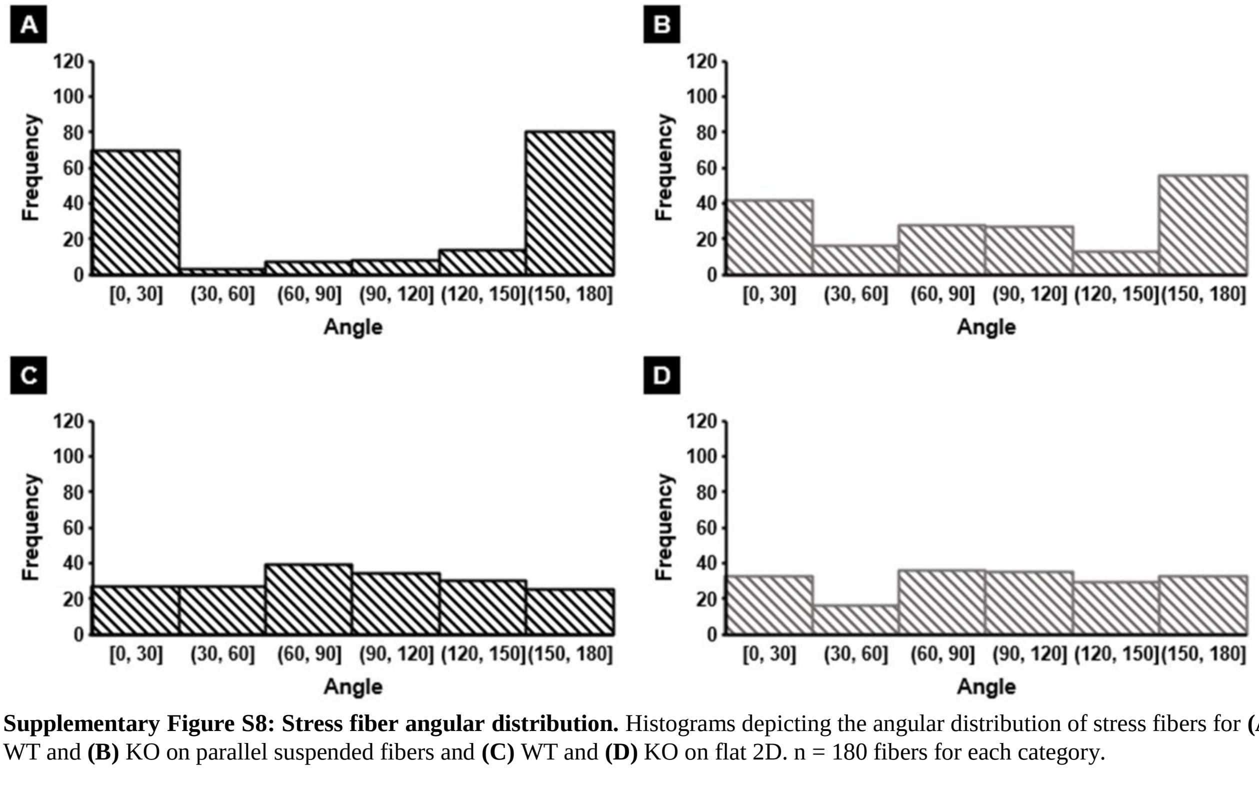

Supplementary Figure S8: Stress fiber angular distribution. Histograms depicting the angular distribution of stress fibers for **(A)** WT and **(B)** KO on parallel suspended fibers and **(C)** WT and **(D)** KO on flat 2D. n = 180 fibers for each category.

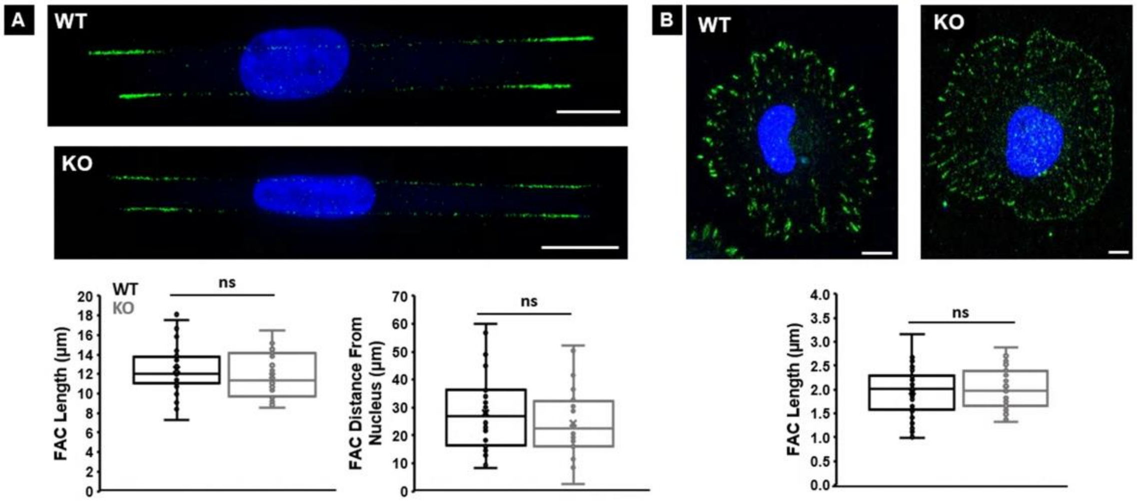

Supplementary Figure S9: Focal Adhesion Cluster (FAC) distribution. Representative fluorescent images of paxillin clustering and associated FAC analysis on (A) 500 nm diameter parallel fibers and (B) flat, 2D for both cell types. Note that the FAC Distance From Nucleus metric is not applicable on flat, 2D surface (see Methods for more details). n values are 30 each for WT and KO cells on both fibers and on flat. All error bars shown represent standard error of mean.

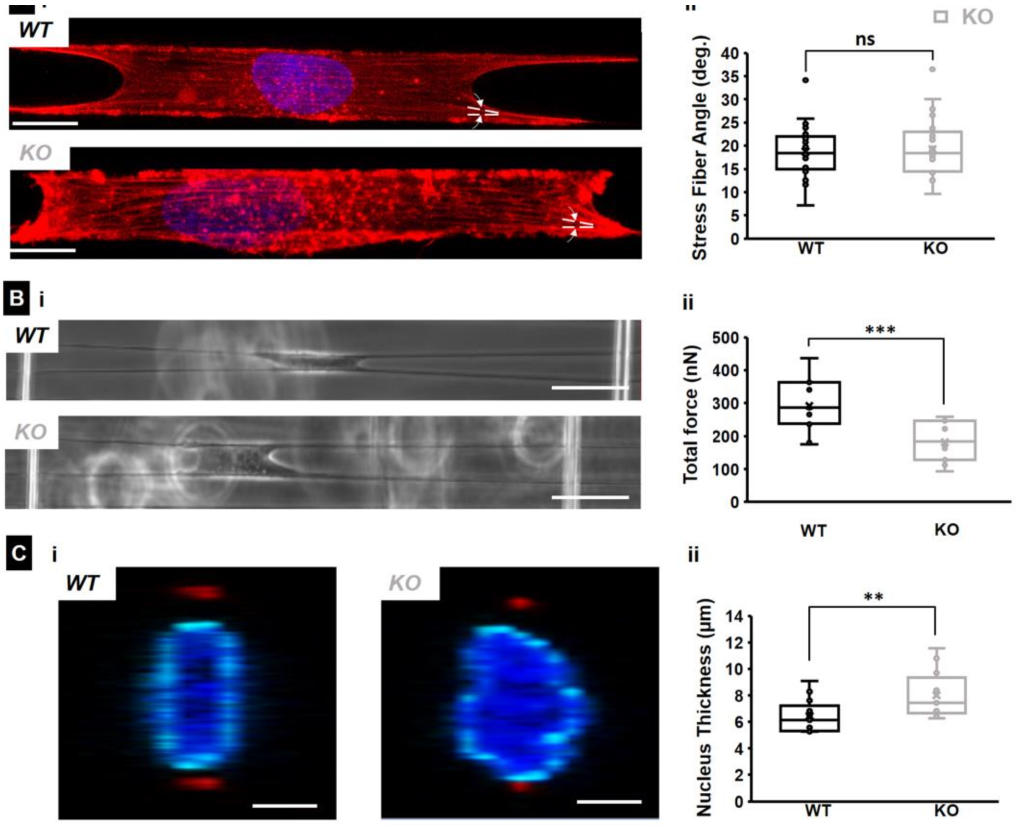

Supplementary Figure S10: IRSp53 KO oral cancer cells show reduced forces and thicker nuclei compared to WT counterparts. (A) Representative fluorescence microscopy images of (i) WT (top) and KO (bottom) cells with f-actin stained in red and (ii) quantification of the stress fiber angles for both cell types on 500 nm diameter fibers. Scale bars are 10 µm. Dotted white lines in the fluorescent images depict the stress fiber angles. n values are 24 and 25 for the KO and WT cells respectively. (B) Representative phase images of (i) WT (top) and KO (bottom) cells exerting forces by pulling on suspended fibers and (ii) quantification of the forces exerted for both cell types on 500 nm diameter fibers. Scale bars are 50 µm. n values are 15 for both cell types. (C) Representative confocal images of (i) WT and KO nucleus cross-section (yz plane) on 500 nm diameter suspended fibers and (ii) quantification of the nucleus thickness. In the confocal images, the nucleus is in blue, the nuclear envelope is in cyan and the cross-section of the suspended fibers is in red. The yellow dotted lines depict the nucleus width. n values are 15 for both cell types. All error bars shown represent standard error of mean.

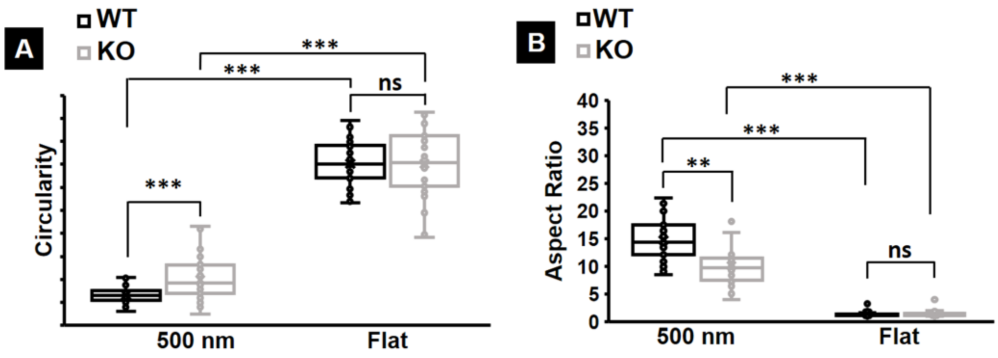

Supplementary Figure S11: Quantification of average morphology metrics during migration. Average (A) circularity and (B) aspect ratio of migrating WT and IRSp53 KO cells on 500 nm diameter suspended fibers and on flat, 2D surface. n for cells on fibers is 35 for both categories and on fibers is 30 for both categories. All error bars shown represent standard error of mean.

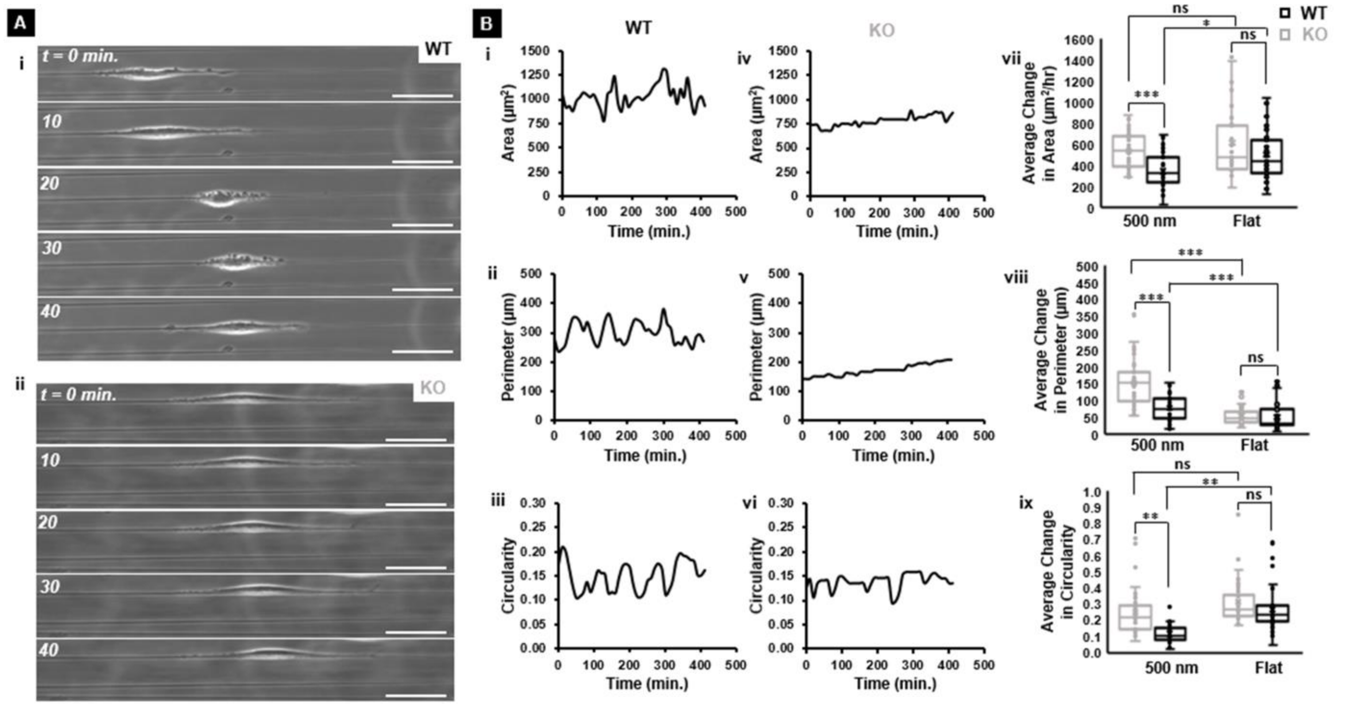

Supplementary Figure S12: Quantification of the morphology changes during migration. (A) Representative phase images showing the change in cell shape during migration for (i) WT and (ii) KO cells. All scale bars are 50 µm. (B) Representative area, perimeter and circularity profiles for (i-iii) WT cells and (iv-vi) KO cells on 500 nm diameter suspended fibers. Quantification of the (vii) average change in area, (viii) average change in perimeter, and (ix) average change in the circularity of WT and KO cells on both 500 nm diameter suspended fibers and flat, 2D surfaces. n values are 35 for both cell types on the 500 nm diameter suspended fiber and 30 for both cell types on the flat surface respectively. All error bars shown represent standard error of mean.

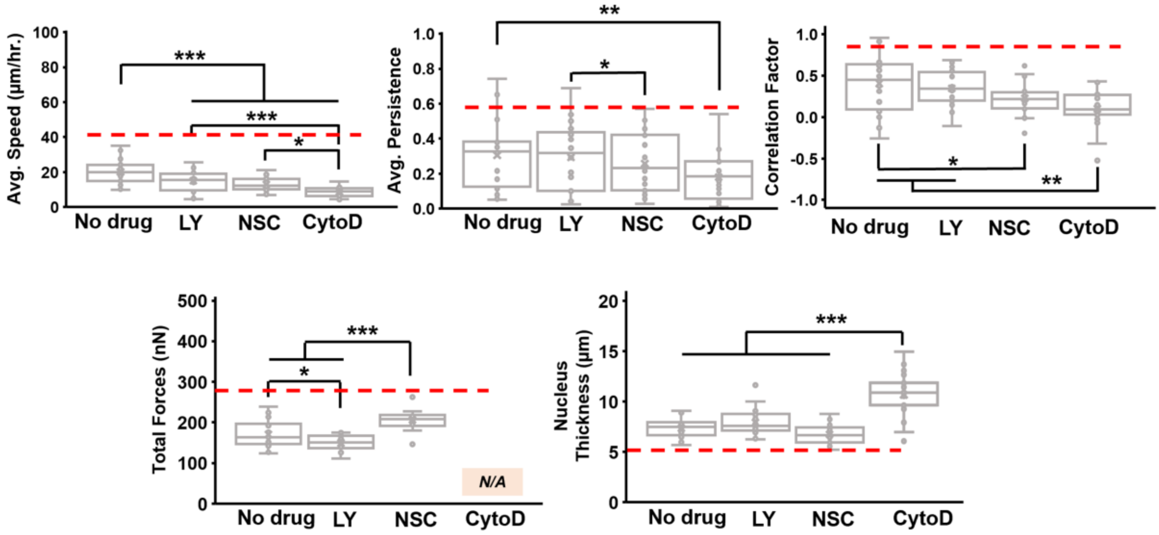

Supplementary Figure S13: Pharmacological studies on IRSp53 KO cells. KO cells treated with LY294002 (PI-3 kinase), NSC23766 (Rac1), and Cytochalasin D (Actin). Red dashed lines represent the IRSp53 WT data without any drug treatment. n = 35 for the control case and n = 25 for each of the drug cases. All error bars shown represent standard error of mean.

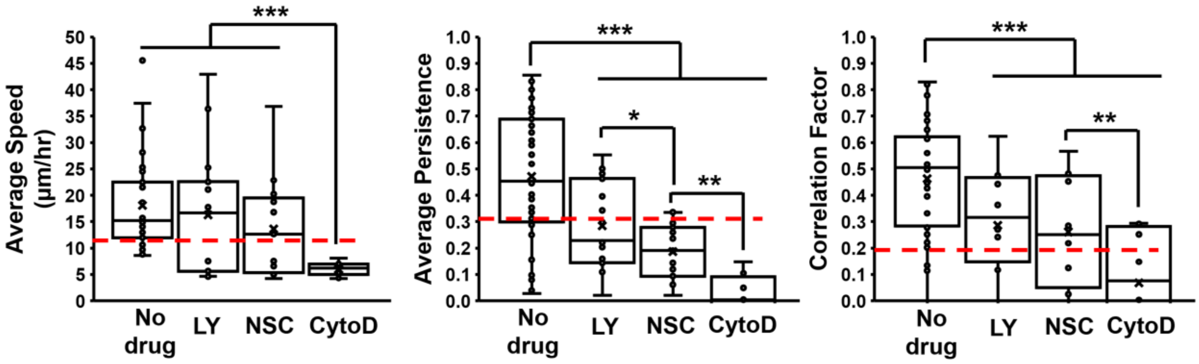

Supplementary Figure S14: Pharmacological studies on IRSp53 WT cells on flat 2D surface. WT cells treated with LY294002 (PI-3 kinase), NSC23766 (Rac1), and Cytochalasin D (Actin). Red dashed lines represent the IRSp53 KO data on flat substrate without any drug treatment. n = 35 for the control case and n = 15 for each of the drug cases. All error bars shown represent standard error of mean.

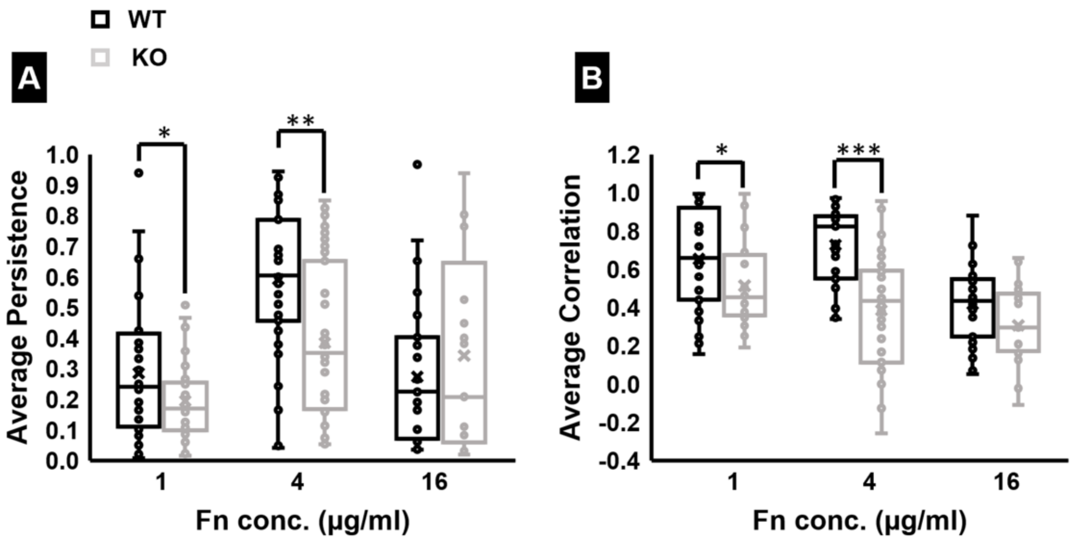

Supplementary Figure S15: Migration dynamics for different fibronectin concentrations. (A) Average persistence, and (B) correlation between nucleus and centroid motion for WT and KO cells on 500 nm diameter fiber. n = 28 for all conditions and both cell types.

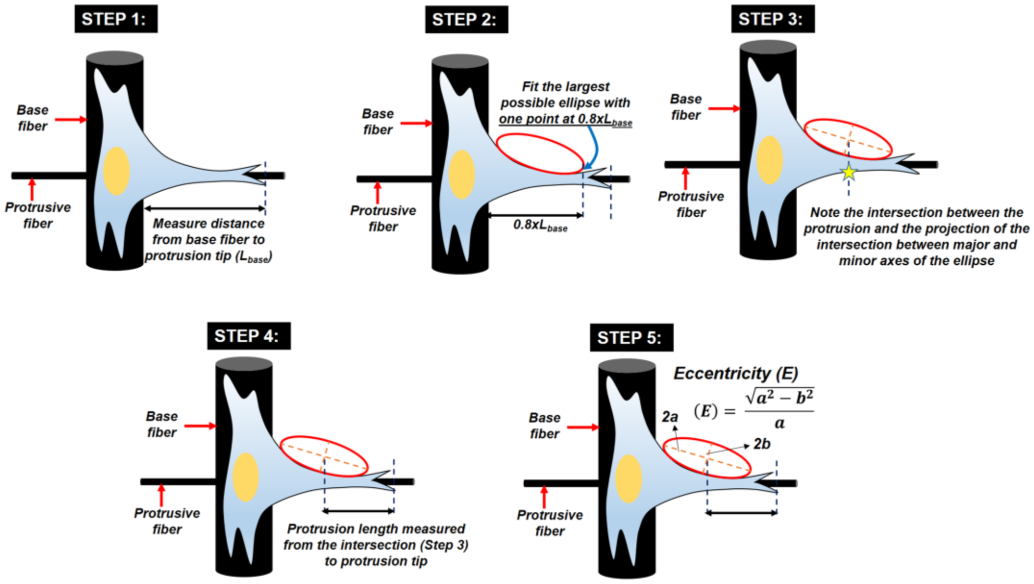

Supplementary Figure S16: Protrusion measurements on suspended fiber networks. Schematic showing stepwise how the protrusion length and eccentricity are measured for single cells on our suspended fiber networks.
